# FNDC5 prevents oxidative stress and neuronal apoptosis after traumatic brain injury through SIRT3-dependent regulation of mitochondrial quality control

**DOI:** 10.1038/s41419-024-06748-w

**Published:** 2024-05-27

**Authors:** Yufeng Ge, Xun Wu, Yaning Cai, Qing Hu, Jin Wang, Shenghao Zhang, Baocheng Zhao, Wenxing Cui, Yang Wu, Qiang Wang, Tian Feng, Haixiao Liu, Yan Qu, Shunnan Ge

**Affiliations:** 1 Department of Neurosurgery, Tangdu Hospital, Fourth Military Medical University, Xi’an, 710038 Shaanxi China; 2 Department of Ambulant Clinic, Political Work Department of People’s Republic of China Central Military Commission, Beijing, China

**Keywords:** Post-traumatic stress disorder, Brain injuries

## Abstract

Mitochondrial dysfunction and oxidative stress are important mechanisms for secondary injury after traumatic brain injury (TBI), which result in progressive pathophysiological exacerbation. Although the Fibronectin type III domain-containing 5 (FNDC5) was reported to repress oxidative stress by retaining mitochondrial biogenesis and dynamics, its possible role in the secondary injury after TBI remain obscure. In present study, we observed that the level of serum irisin (the cleavage product of FNDC5) significantly correlated with the neurological outcomes of TBI patients. Knockout of FNDC5 increased the lesion volume and exacerbated apoptosis and neurological deficits after TBI in mice, while FNDC5 overexpression yielded a neuroprotective effect. Moreover, FNDC5 deficiency disrupted mitochondrial dynamics and function. Activation of Sirtuin 3 (SIRT3) alleviated FNDC5 deficiency-induced disruption of mitochondrial dynamics and bioenergetics. In neuron-specific SIRT3 knockout mice, FNDC5 failed to attenuate TBI-induced mitochondrial damage and brain injuries. Mechanically, FNDC5 deficiency led to reduced SIRT3 expression via enhanced ubiquitin degradation of transcription factor Nuclear factor erythroid 2-related factor 2 (NRF2), which contributed to the hyperacetylation and inactivation of key regulatory proteins of mitochondrial dynamics and function, including OPA1 and SOD2. Finally, engineered RVG29-conjugated nanoparticles were generated to selectively and efficiently deliver irisin to the brain of mice, which yielded a satisfactory curative effect against TBI. In conclusion, FNDC5/irisin exerts a protective role against acute brain injury by promoting SIRT3-dependent mitochondrial quality control and thus represents a potential target for neuroprotection after TBI.

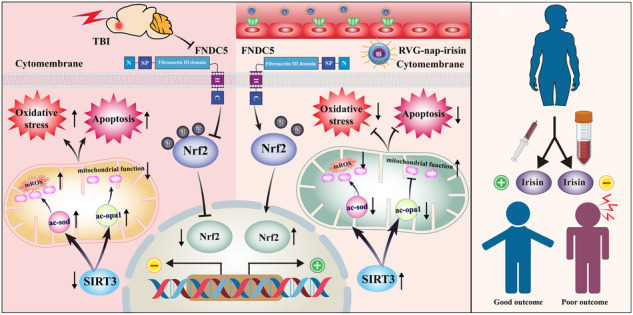

## Introduction

Traumatic brain injury (TBI) is the leading cause of adult disability resulting from a head impact or external force [1, 2]. Current evidence suggests that ~43% of TBI patients suffer long-term disability [3, 4]. Secondary brain injuries after TBI contribute to progressive pathophysiological exacerbation and unsatisfactory neurological outcomes to a large extent [5, 6]. It has been established that secondary brain injuries are characterized by pathological changes, including abnormal mitochondrial activity, oxidative stress, inflammatory events, glutamate toxicity, and brain edema [7–10]. Unfortunately, few effective treatments are currently available for clinical practice.

Fibronectin type III domain-containing protein 5 (FNDC5) is a glycosylated transmembrane protein containing two fibronectin domains and a hydrophobic domain [11**–**14]. Cleavage of FNDC5 results in the formation of irisin. Although the roles of FNDC5/irisin in metabolic disorders have been documented, it is widely thought that these proteins have various functions in the central nervous system (CNS) [11, 15, 16]. In this respect, Hashemi [17] et al. found that FNDC5 knockdown in neuroblasts could impair their differentiation into mature neurons, suggesting a critical role of FNDC5 in neuronal development. Mychael [18] et al. found that FNDC5/irisin could restore memory and synaptic plasticity in AD mouse models. In addition, many studies confirmed that boosting brain levels of FNDC5 could confer neuroprotective effects. The protective effects of FNDC5/irisin on cerebral ischemia stroke and multiple sclerosis have been demonstrated recently [19, 20]. However, the possible role of FDNC5 after acute TBI needs further research.

The mitochondrion is the main source of energy supply for cellular activities. Dysregulated mitochondrial bioenergetics leads to ineffective ROS production, insufficient ATP energy supply, and abnormal mitochondrial cytochrome c release [21**–**24]. There is an increasing consensus that regulating mitochondrial quality control (including mitochondrial dynamics, bioenergetics, and mitophagy) is vital in protecting against acute/chronic brain injury [25**–**28]. FNDC5/Irisin was reported to repress oxidative stress by retaining mitochondrial biogenesis and dynamics [29]. Another study demonstrated that exogenous irisin alleviates liver I/R injury by promoting mitochondrial biogenesis and relieving oxidative stress [30]. In addition, it has been reported that damage to mitochondrial quality control contributes to CNS disease while boosting either brain or peripheral FNDC5/irisin levels attenuates synaptic and memory impairments in AD mouse models, suggesting a potential neuroprotective role. Nonetheless, whether FNDC5 affects mitochondrial quality control process after TBI remains unclear.

To bridge this knowledge gap, we investigated clinical evidence concerning the relationship between serum irisin levels and TBI patient outcomes. Given that mitochondrial injury and oxidative stress may mediate secondary injury to perilesional tissue after TBI, the present study examined the effect of FNDC5 on the secondary injury of perilesional tissue after TBI and confirmed that reducing mitochondrial dysfunction plays a crucial role in this process. We provided hitherto undocumented evidence that FNDC5 regulates the expression of SIRT3 (a critically important deacetylase in mitochondria) by modulating the protein stability of transcription factor Nuclear factor erythroid 2-related factor 2 (NRF2). Finally, we assessed the therapeutic effects of brain-targeted irisin supplementation delivered with engineered RVG29-conjugated nanoparticles.

## Materials and methods

### Patients

The study participants were adults aged 18–80 with mild, moderate, or severe TBIs from the emergency room. All patients with a history of disabling neurological conditions or severe systemic diseases, such as uremia, cirrhosis, or cancer, were excluded. The Glasgow Outcome Scale (GOS) scores were used to evaluate the neurological function of TBI patients six months after surgery (The specific details of GOS rating can be found in Supplementary Material Table 1). Scores 1–3 and 4–5 represented unfavorable and favorable outcomes, respectively. Ethics approval (No. K201907-03) was received from the Ethics Committee of Tangdu Hospital, Fourth Military Medical University.

### Animals and ethics approval

All animal care and experimental procedures described in the paper were approved by the Animal Care group and by the Ethics Committee of the Fourth Military Medical University and in compliance with ethical guidelines. The Animal Center of the Fourth Military Medical University provided experimental C57BL/6 mice (8–12 weeks old, 20–25 g). Mice carrying SIRT3-floxed alleles (SIRT3^f/+^) were obtained from the Jackson Laboratory. FNDC5 knockout mice and Map2-CreERT2 mice were purchased from the Shanghai Model Organisms Center. Mice lacking SIRT3 in neurons were generated by crossbreeding SIRT3^f/f^ mice with Map2-CreERT2 transgenic mice, followed by intraperitoneal injection of 5 doses of tamoxifen, 75 mg each time, at an interval of 5 days. Mice were housed in a pathogen-free environment at the Neurosurgery Laboratory of Tang Du Hospital.

### Stereotaxic injection of the adeno-associated virus

The AAV9 virus expressing FNDC5 (AAV-FNDC5) or AAV9-CON323 (AAV-Ctrl) from Genchem (Shanghai, China) was used for stereotaxic injection. Briefly, FNDC5 sequence was inserted into EcoRI and BamHI sites of the hSyn promoter-MCS-EGFP-3FLAG-SV40 PolyA (GV466) AAV vector. The mice were anesthetized with 2% pentobarbital sodium for viral injections in stereotactic racks. Cortex injections were performed in the right hemisphere (ipsilateral to the treatment) as follows: anterior–posterior 1.5 mm, medial-lateral 1.5 mm, and dorsal-ventral 1.00 mm. The injected virus contained 1.5 μl of 1 × 10^9^ TU/ml AAV suspension, which was injected at a rate of 0.2 μl/min. The needle was withdrawn over the course of 10 min. As described above, mice were subjected to brain trauma 14 days after lentivirus injections.

### TBI surgery

The TBI mouse model was established using a controlled cortical impact (CCI) device. Briefly, an intraperitoneal injection of 2% pentobarbital sodium was employed for the anesthesia of the experimental mice, and the stereotactic device was used to fix the mouse heads. Then, a 2-mm diameter drill was applied to perform a craniotomy above the right parietal bone window (1.5 mm behind the bregma and 1.5 mm backward the midline). The traumatic injury was induced using a TBI impactor with a flat metal tip using the following parameters: speed of 3 m/s, contact time of 0.2 s and depth of 1.5 mm. The TBI mice were placed next to a heater until they regained consciousness before returning to their home cage.

### ELISA

Blood samples were obtained from patients with TBI. The anticoagulated blood was centrifuged (1500 rpm, 15 min) to obtain the serum for ELISA analysis. Commercial ELISA Kits (ml037372, mlbio, RAG018R, BioVendor R&D, and NBP3-08117, Novus) were used to test and validate serum irisin levels, and the irisin-specific Luminex bead-based multiplex detection system (Merck Millipore) was further employed to detect human serum irisin. Detailed irisin assays were performed according to the kit instructions.

### Immunofluorescence and TUNEL staining

Coronal brain sections were stained following a published protocol. Briefly, the brain sections (15 μm) were incubated for 30 min in PBS with 5% goat serum (Gibco) and 0.1% Triton X-100. The sections were probed with anti-NeuN (ABN78, Millipore; diluted 1:500) primary antibodies overnight at 4 °C, followed by incubation with a secondary goat anti-rabbit antibody (ab150077, abcam, diluted 1:1000) for 50 min at room temperature. Nuclei were stained with DAPI (62248, 1:1000, Invitrogen) for 15 min at room temperature, and the sections were evaluated with an A1 Si confocal microscope (Nikon) and imaged.

Apoptosis was assessed by TUNEL assays (11767305001 and 11966006001, Roche) following the provided instructions. The sections were evaluated by confocal microscopy as outlined above, and the degree of apoptosis was calculated as the ratio of TUNEL-stained cells to DAPI-stained cells.

### Quantification of lesion volume

Small-animal MRI (Bruker) scans were used to evaluate the lesions. The total lesion volume was assessed through T2-weighted imaging (T2). Medical image processing, analysis, and visualization (MIPAV) software was used to outline the volumes, which were then calculated by multiplying the sum of the volume by the distance between sections (0.7 mm-thick).

### Measurement of cerebral water content

The ratios between wet and dry weight were used to quantify the cerebral water content, as previously described. The brains were removed, weighed (wet weight) and oven-dried at 95–100 °C for 72 h, followed by reweighing (dry weight). The brain water content (%) was calculated as brain water content (%) = (wet weight − dry weight)/wet weight × 100%.

### Neurobehavioral tests

#### The grid walking test

Experimental mice were attached to an elevated metal wire with an opening in the center and videotaped from below the wire for 1 min. A blinded investigator analyzed the videotape to determine how many steps were taken and the number of foot faults. Mouse foot faults were measured by determining whether their impaired forepaws or hind paws fell through the grid due to misplacement.

#### Adhesive removal test

A piece of 2 mm × 3 mm adhesive tape was attached to the left forepaw (impaired side), and the time taken until the mouth made contact with the paw was determined. The time until the removal of the tape from the paw was also recorded. The observation time was 120 s.

#### Cylinder test

Mice were placed in 15-cm-high and 9-cm-diameter transparent cylinders and filmed from the top for 8 min. The videos were analyzed in slow motion, and recordings were made of paw use (left, right, both) during the first contact with the cylinder wall after rearing and when exploring laterally. Mouse lines with uninjured forepaws typically do not show any preference for either forepaw, while injury results in decreased use of the impaired paw. Forepaw use asymmetry was calculated with the following equation: contralateral paw use = (right-left)/(left + right + both) × 100%.

#### Morris water maze

A 90-cm diameter pool with a submerged square platform (11 × 11 cm^2^) was filled with opaque water containing nontoxic paint. During the learning stage of the experiment, the mouse was placed in different parts of the pool and allowed to swim for 60 s or until finding the platform. The interval between placement in the pool and finding the platform (escape latency) was recorded as a measure of ‘spatial learning’. The mouse was then allowed to stay on the platform or was placed on the platform for 10 s if unable to locate the platform within 60 s. Spatial cues were provided. Four trials were conducted per day for five consecutive days. In the memory assessment stage of the experiment, the platform was removed, and the mouse was again placed in the pool for a single 60-s trial.

### DHE staining

ROS levels were measured by dihydroethidium (DHE) staining. Brain sections (15-µm thick) were incubated with 3 µM DHE (Yeasen, 50102ES02) for 30 min and evaluated by laser-scanning confocal microscopy.

### Transmission electron microscopy

Mouse brains were placed in chilled Hanks’ Balanced Salt Solution after removal. Tissue samples (1 × 2 mm^2^) were fixed with 4% glutaraldehyde overnight at 4 °C, followed by treatment with 1% osmium tetroxide (OsO_4_) for 1 h at 4 °C, dehydration in an ethanol gradient, and embedding in resin. The samples were then trimmed and sectioned using an ultramicrotome and placed on 200-slot grids coated with polyvinyl alcohol ester. Cell morphology and mitochondria were evaluated using a JEM-1400 electron microscope (JEOL) coupled with a camera (Olympus).

### Cell culture and treatment

Mouse neuronal HT-22 cells were purchased from Procell Life Science & Technology (CL-0697, Wuhan, China) and seeded at a density of 2 × 10^6^ cells in 6 cm dish with DMEM (Gibco, USA) containing 10% fetal bovine serum (FBS, Gibco) and 1% penicillin-streptomycin (Corning, Glendale, AZ, USA). Cells were grown in a humidified incubator at 37 °C and 5% CO_2_ and were subcultured thrice a week. CRISPR/Cas9 gene editing was used to knock out FNDC5 in HT22 cells. Briefly, after transduction with Lenti-CAS9 virus for three days, cells were incubated with 200 µg/ml G418 for an additional seven days. After cloning sgRNAs into Lenti-CAS9-puro, cells were incubated for seven days with 200 µg/ml G418 and 1 µg/ml puromycin to select positive clones. Targeted sequences for the HT22 mouse cell line: (5ʹ-GCTCTTCAAGACCCCACGCG-3ʹ), (5ʹ-TGCCGGACGGTCACGTTCAC-3ʹ) and (5ʹ-AGAATATATCGTCCATGTGC-3ʹ).

### Western blotting

Brain tissue or collected cells were ultrasonically homogenized in cold lysis buffer with protease and phosphatase inhibitors. Protein levels were determined using a BCA Protein Assay kit (Thermo Scientific, MA, USA, UA276918). SDS–PAGE was used to separate the extracted proteins, which were then transferred to PVDF membranes (Millipore). After incubation with 5% nonfat milk, the blots were probed with the following primary antibodies: anti-FNDC5 (1:1000; ab174833, Abcam); anti-SIRT3 (1:1000; D22A3, Cell Signaling Technology); anti-Bcl2 (1:1000; gtx100064, Gene Tex); anti-Bax (1:1000; 50599-2-Ig, ProteinTech); anti-Nrf2 (1:1000; 16396-1-AP, ProteinTech); and anti-β-actin (1:3000; WL01372, Wanleibio), the primary antibodies were used to incubate membranes overnight at 4 °C. After incubation with HRP-conjugated secondary antibodies (1:5000; AS003, AS014, ABclonal)for 2 h at room temperature, the bands were visualized by chemiluminescence using a Bio-Rad Imaging System (Bio-Rad) and analyzed with ImageJ software.

### Analysis of mitochondria morphology and function

The morphology and function of the mitochondria were examined as previously described [31]. For fluorescence labeling, the treated cells were incubated with 10 nM MitoTracker Red (M7512, Life Technologies), and the mitochondria were examined under fluorescence microscopy. Functional assessments included measurements of the mitochondrial membrane potential (MMP) and mitochondrial ROS production. The cells were exposed to 10 nM JC-1 (C2006, Beyotime) and 5 nM MitoSOX (M36009, Invitrogen) for 30 min. The images were captured with a fluorescence microscope, and the fluorescence intensity was determined by ImageJ software.

### Preparation of RVG-Nap-irisin and drug treatment

Nanoparticles were synthesized using the nanoprecipitation technique [32]. PLGA20K (10 mg), PLGA20K-mPEG2K (10 mg), and PLGA20K-PEG5K-MAL (5 mg) in 1 ml of chloroform were fully dissolved by sonication (about 30 s) and placed at 4 °C until use. Irisin was reconstituted using ultra-pure water, which included 1% polyvinyl alcohol as a reagent, with prior pre-cooling. The resulting protein solution had a concentration of 10 μg/ml and was utilized at a temperature of 4 °C. The organic phase solution was introduced into the endotoxin-free aqueous phase and agitated at a temperature of 4 °C for a duration of 5 h, until complete evaporation of chloroform occurred. Nanoparticles were concentrated by ultrafiltration (washing three times) with a 100 kDa cut-off molecular weight ultrafiltration tube. The resulting product was then dispersed in PBS (containing 0.1% EDTA), followed by the addition of RVG peptide solution for the intended preparation. The mixture was shaken overnight at a temperature of 4 °C, and subsequent ultrafiltration was performed using a 100 kDa cut-off molecular weight ultrafiltration tube, which was washed three times. The resulting products were redispersed in PBS and stored at a temperature of 4 °C until needed. Single doses of irisin and RVG-Nap-irisin (100 µg/kg) were given by intravenous injection every day after surgery.

### Determination of ATP, MDA, and activities of antioxidant enzymes

The mice were sacrificed 24 h after TBI; their tissues were harvested, lysed in lysis buffer, and homogenized on ice for 30 min. The supernatants were obtained after centrifuging samples for 10 min at 4 °C (12,000 rpm). ATP levels (BC0300, Solarbio, China), Malondialdehyde (MDA) (S0131S, Beyotime, Jiangsu, China), NAD + (ab65348; Abcam), SOD (S0101 M, Beyotime, Jiangsu, China) and activities of oxidative stress-related enzymes were measured as instructed by the corresponding commercial kits.

### Statistical analysis

Continuous variables that exhibited a nonparametric distribution were expressed as medians with interquartile ranges and as the means ± standard deviations (SD) if normally distributed (datas of patients). Data of animals in this research were expressed as mean ± standard error of the mean (SEM). Categorical data were expressed as frequencies and percentages. Multivariate logistic regression was used to identify independent risk factors for unfavorable six-month outcomes of patients. Spearman’s rank correlations were used for univariate correlation analysis. Unpaired two-tailed *t* tests were used for comparing two independent groups, and one-way ANOVA was used for multiple group comparisons. Two-way repeated measures ANOVA was used for multiple comparisons of animal behavioral data. The significance of comparisons between the two groups was determined by Student’s t-test (two-tailed). A *P* value < 0.05 was statistically significant.

## Results

### Association between serum irisin levels and outcomes in TBI patients

**Fig. 1:**
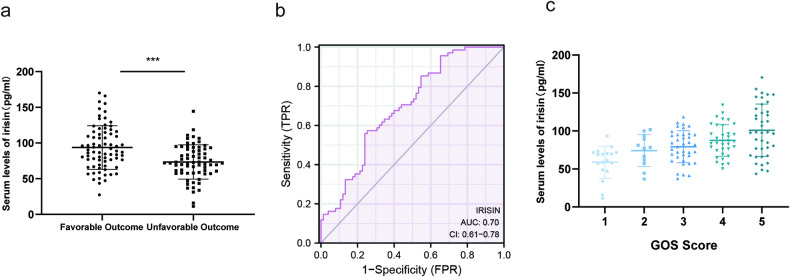
Association of serum irisin levels with outcomes in TBI patients. **a** Serum irisin levels were decreased in patients with poor outcomes compared to those with good outcomes (*P* < 0.001). **b** ROC analysis of the discriminative ability of serum irisin levels for risk of poor outcome in patients with TBI. **c** Linear correlation between serum irisin levels and short-term neurological outcome assessed using GOS scores of patients with TBI. Significance was determined by Student’s *t* test (**a**) and one-way ANOVA (**c**). Values are presented as the mean ± SD.

The present study included 143 patients with TBI, and their baseline characteristics are listed in Table 1. Three distinct human irisin ELISA kits and the Luminex-based multiplex detection system specifically designed for irisin were collectively validated, revealing a statistically significant elevation in serum irisin concentrations among patients with favorable outcomes (*P* < 0.001; Fig. 1a, and Fig S1a–c). ROC curve analyses showed an area under the ROC curve of irisin was 0.7 (Fig. 1b). Spearman correlation analysis between serum irisin levels and outcomes defined by six-month GOS scores showed a significant positive correlation (*P* < 0.001; Fig. 1c). Univariate analysis showed that increased serum irisin levels (OR = 0.97, 95% CI: 0.95–0.99, adjusted *P* = 0.002; Table 1) were an independent predictor of favorable outcomes.

**Table 1:** Characteristics of the TBI patients.

	Level	Favorable outcome *n* = 75		Unfavorable outcome *n* = 68	Crude OR(95%CI)		Crude *P* value	Adjusted OR(95%CI)	Adjusted *P* value
sexuality (%)	male	60 (80.0)	48 (70.6)			REF.			
	famale	15 (20.0)	20 (29.4)			1.67 (0.77–3.6)	0.193		
Injury mechanism (%)	strike	36 (48.0)	35 (51.5)			REF.			
	accident	2 (27)	5 (74)			2.57 (0.47–14.14)	0.277		
	fall	35 (46.7)	24 (35.3)			0.71 (0.35–1.42)	0.326		
	other	2 (27)	4 (59)			2.06 (0.35–11.96)	0.422		
Abnormal pupil reaction (%)	no responses	36 (48.0)	35 (51.5)			REF.			
	slow	4 (53)	9 (13.2)			2.31 (0.65–8.21)	0.194	2.14 (0.26–17.39)	0.476
	sensitive	35 (46.7)	24 (35.3)			0.71 (0.35–1.42)	0.326	0.39 (0.14–1.09)	0.071
Coagulopathy (%)	no	38 (80.9)	31 (75.6)			REF.			
	yes	9 (19.1)	10 (24.4)			1.36 (0.49–3.77)	0.552		
IRISIN1 (mean (SD))		93.52 (28.22)	72.96 (22.51)			0.97 (0.95–0.98)	<0.001	0.97 (0.95–0.99)	0.002
Age (mean (SD))		53.27 (13.21)	55.26 (13.01)			1.01 (0.99–1.04)	0.362	1.05 (1–1.09)	0.037
GCS (mean (SD))		11.04 (2.58)	8.60 (3.04)			0.73 (0.63–0.84)	<0.001	0.69 (0.57–0.82)	<0.001
PT (mean (SD))		11.36 (0.89)	11.87 (1.78)			1.37 (0.98–1.93)	0.066	1.06 (0.66–1.69)	0.817
PTA (mean (SD))		96.86 (14.76)	91.79 (19.65)			0.98 (0.96–1.01)	0.133		
INR (mean (SD))		1.01 (0.09)	1.05 (0.17)			11.35 (0.44–295.61)	0.144		
Fib (mean (SD))		2.84 (0.99)	2.61 (1.29)			0.83 (0.59–1.17)	0.29		
RBC (mean (SD))		4.06 (0.78)	4.11 (0.67)			1.09 (0.69–1.73)	0.709		
HCT (mean (SD))		46.84 (30.45)	37.38 (4.92)			0.95 (0.87–1.04)	0.279		
PLT (mean (SD))		172.78 (69.21)	179.33 (69.24)			1(1–1.01)	0.577		
Hb (mean (SD))		134.51 (19.78)	129.38 (22.25)			0.99 (0.97–1.01)	0.206		
ALB (mean (SD))		40.10 (5.94)	41.30 (5.87)			1.04 (0.98–1.1)	0.235		
AST (mean (SD))		41.41 (37.22)	53.72 (33.34)			1.01 (1–1.02)	0.059	1(0.98–1.01)	0.73

### FNDC5 expression determined lesion volume, brain edema, and neurological deficits after TBI

#### Knockout of FNDC5 increased lesion volume, worsened brain edema, and exacerbated neurological deficits after TBI

**Fig. 2:**
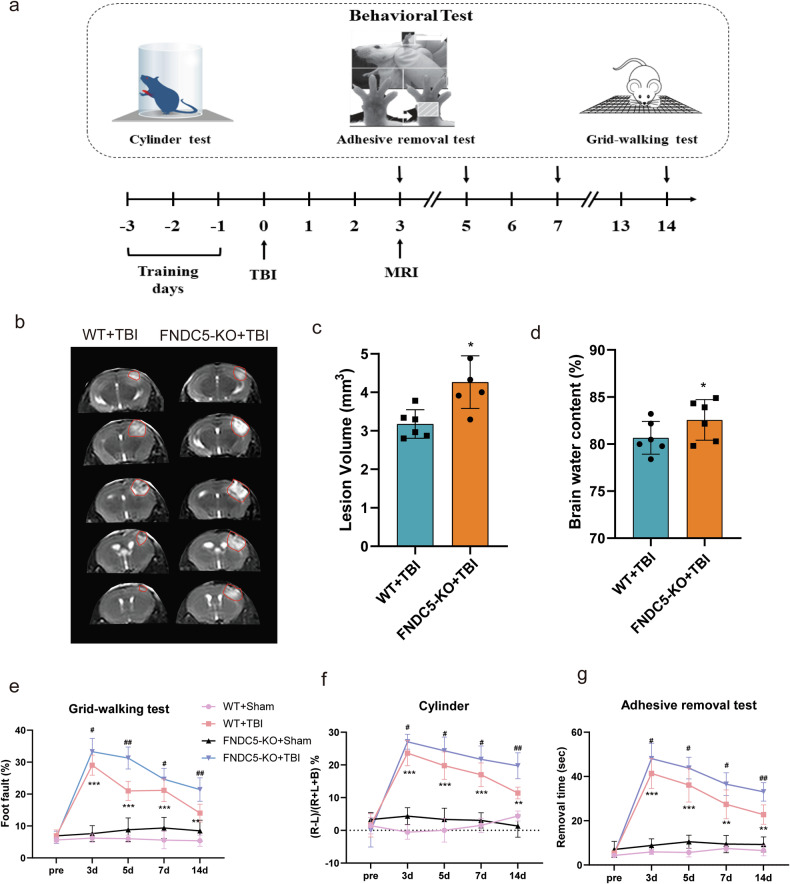
Knockout of FNDC5 increases lesion volume, worsens brain edema, and aggravates neurological deficits after TBI. **a** Schematic of MRI test and behavioral studies. **b** MRI scan of the brain in WT + TBI group and FNDC5-KO + TBI group; *n* = 6 for each group. **c** Quantification of lesion volume of MRI. **d** measurement of brain water content. *P < 0.05, **P < 0.01 and ***P < 0.001 vs. WT + TBI group. **e**–**g** Several behavioral tests at different time points after TBI as measured by the grid-walking test (**e**), cylinder test (**f**) and adhesive removal test (**g**) (*n* = 9 for each group). Significance was determined by Student’s *t* test (**c**, **d**) and two-way repeated ANOVA (**e**–**g**) with Bonferroni post hoc tests. *P < 0.05, **P < 0.01 and ***P < 0.001 vs. WT+Sham group, ^#^*P* < 0.05 and ^##^*P* < 0.01 vs. WT + TBI group. Values are presented as the mean ± SEM.

FNDC5 knockout mice were established to determine the potential role of FNDC5 in brain injury after TBI. The MRI examination was performed on day 3, and animal behavioral tests were performed on days 3-14 after TBI (Fig. 2a). MRI was conducted to measure lesion volume. The results showed that knockout of FNDC5 significantly increased TBI-induced lesion volumes (Fig. 2b, c). Then, the brain water content in each group was determined. Compared with the WT mice group, TBI-induced brain edema in the FNDC5 KO mice group was more severe (Fig. 2d). Next, behavioral tests, including the cylinder, adhesive, and grid walking tests were performed (Fig. 2e–g). As shown in Fig. 2e, the percentage of foot faults in the grid-walking experiment after TBI was higher in the FNDC5 KO mice group compared to the WT mice group. In the cylinder experiment, the FNDC5 KO mice showed more bias than WT mice after TBI (Fig. 2f). Similar results were observed in the adhesive removal test (Fig. 2g). Overall, knockout of FNDC5 increased lesion volume, worsened brain edema and aggravated neurological deficits after TBI.

#### Overexpression of FNDC5 reduces lesion volume, attenuates brain edema and improves neurological function after TBI

To investigate whether FNDC5 could protect against brain injury after a traumatic brain injury, FNDC5 was overexpressed in neurons by injecting the AAV9 virus expressing FNDC5 using the syn promoter. MRI results indicated that TBI-induced brain lesion volume was significantly reduced after FNDC5 overexpression, suggesting that FNDC5 alleviated gross brain tissue damage after TBI (Fig. S2a, b). Overexpression of FNDC5 attenuated brain edema after TBI in mice (Fig. S2 c). Moreover, we found that the neurological function was improved in the FNDC5-overexpressing mice group. As shown in Figure S2 d-f, FNDC5 overexpression improved the behavioral function of animals in the grid-walking experiment, cylinder experiment and adhesive removal tests. Taken together, these results suggest that overexpression of FNDC5 promoted recovery in traumatic brain injury.

### Overexpression of FNDC5 can suppress oxidative stress and reduce neural apoptosis after TBI

**Fig. 3:**
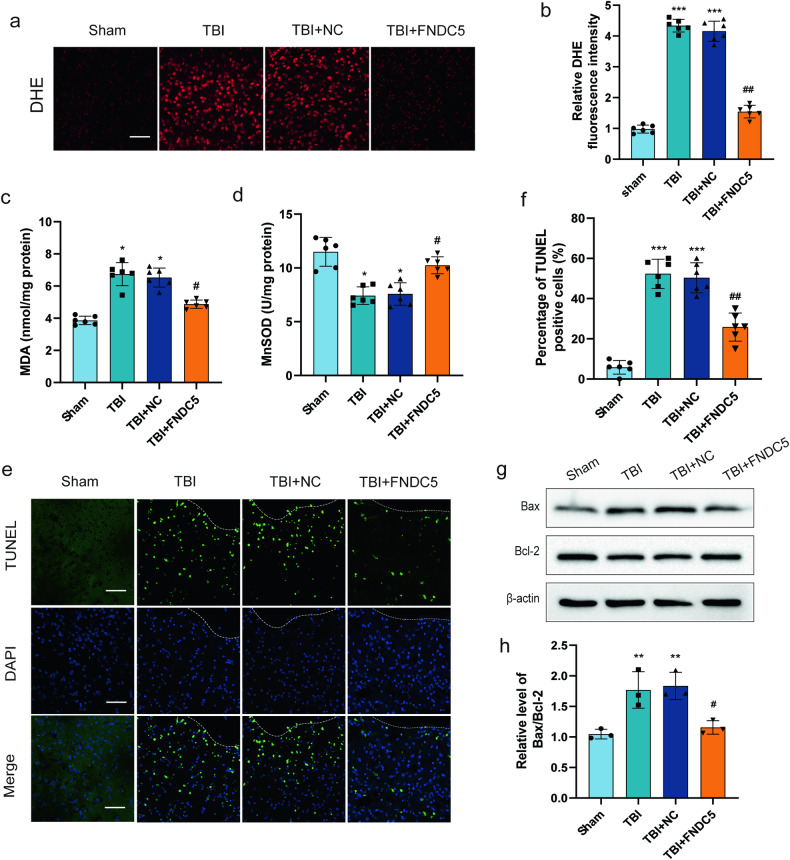
FNDC5 suppresses oxidative stress and reduces neural apoptosis after TBI. **a**, **b** Representative DHE staining images and the quantitative results (*n* = 6 for each group). Scale bar: 200 μm. **c** The effects of FNDC5 on MDA levels. **d** The effects of FNDC5 on mitochondrial MnSOD activity. **e**, **f** Representative images and statistical analysis of TUNEL staining of the perilesional cortex after TBI. Scale bar: 200 μm. **g**, **h** Representative Western blots and statistical analysis of the levels of Bax and Bcl-2 (*n* = 6 for each group). Significance was determined by one-way ANOVA (**b**–**d**, **f**, **h**) with Bonferroni post hoc tests. *P < 0.05 and **P < 0.01 vs. sham group, ^#^*P* < 0.05 and ^##^*P* < 0.01 vs. TBI + NC group. Values are presented as the mean ± SEM.

Oxidative stress and neural apoptosis are well-established features of secondary brain injury following TBI. In this study, DHE staining showed that TBI increased brain ROS production, and this phenomenon could be reversed by FNDC5 overexpression (Fig. 3a, b). It was also observed that FNDC5 overexpression lowered the increased MDA levels (Fig. 3c) and restored the suppressed MnSOD activity after TBI (Fig. 3d). Evaluation of the effects of FNDC5 on apoptosis through TUNEL staining (Fig. 3e, f) showed increased apoptosis after TBI, which were decreased by FNDC5 overexpression. Consistently, western blotting substantiated that increased FNDC5 reduced the BAX/BCL-2 ratio after TBI (Fig. 3g, h).

### FNDC5 deficiency damages mitochondrial dynamics and bioenergetics, which was reversed by activation of SIRT3

**Fig. 4:**
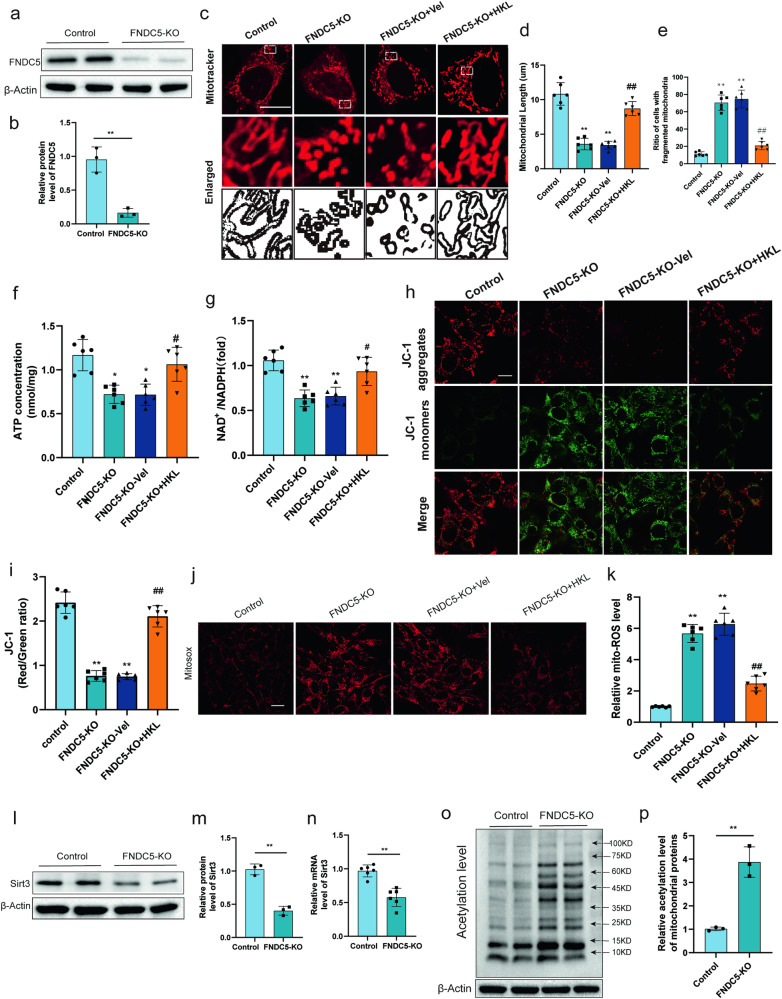
FNDC5 deficiency disrupts mitochondrial dynamics and bioenergetics, and activation of SIRT3 alleviates FNDC5 deficiency-induced damage to mitochondrial dynamics and bioenergetics. **a**, **b** Western blot and statistical analysis of FNDC5 expression. **c** Representative MitoTracker fluorescence images illustrating mitochondrial morphology. Scale bar: 10 μm. **d**, **e** Mitochondrial morphological characteristics were quantified by ImageJ software. **f** ATP content of HT22 cells. **g** NAD^+^/NADH of HT22 cells. **h**, **i** Representative fluorescence intensity of JC-1 staining illustrating the MMP. Scale bar: 20 μm. **j**, **k** Representative MitoSOX fluorescence images of mitochondria-derived ROS. Scale bar: 50 μm (*n* = 6 for each group). **l**, **m** Western blot and statistical analysis of SIRT3 expression. **n** Relative mRNA level of SIRT3. **o** Total acetylation level of mitochondrial proteins. **p** Relative acetylation level of mitochondrial proteins. Significance was determined by Student’s *t* test (**b**, **m**, **n**, **p**) and one-way (**d**–**g**, **i**, **k**) ANOVA with Bonferroni post hoc tests. *P < 0.05 and **P < 0.01 vs. Control group, ^#^*P* < 0.05 and ^##^*P* < 0.01 vs. FNDC5-KO + vel group. Values are presented as the mean ± SEM.

To further study the biological effect of FNDC5, an FNDC5 KO HT22 cell line was created by lentiviral expression of nuclease-active Cas9 and FNDC5 gRNA (Fig. 4a, b). Mitochondrial quality control is recognized as a vital function in mitigating neuronal damage after TBI. The effects of FNDC5 deletion on mitochondrial dynamics, bioenergetics and mitophagy were investigated. Then, the effects of FNDC5 deletion on mitochondrial dynamics, bioenergetics and mitophagy were investigated. Figure 4c–e showed that the mitochondria in the vehicle group appeared as highly interconnected elongated tubules. In contrast, FNDC5 deficiency resulted in spherical and shorter mitochondria, indicating that mitochondrial morphology was damaged in the absence of FNDC5. Next, mitochondrial bioenergetics was also detected. Results showed that ATP production (Fig. 4f), the NAD+/NADH ratio (Fig. 4g), and the mitochondrial membrane potential (Fig. 4h, i), which are indices of mitochondrial bioenergetics, were damaged by FNDC5 deficiency. In addition, the levels of mitochondrial ROS (mtROS) were significantly increased in FNDC5 knockout cells (Fig. 4j, k). The above results showed that the deficiency of FNDC5 negatively affected mitochondrial dynamics and bioenergetics.

Then, we investigated the mechanisms underlying alleviated mitochondrial damage induced by FNDC5 deficiency. We detected the key proteins regulating mitochondrial dynamics (DRP1, MFN1, MFN2, and OPA1), but found no significant change in their protein expressions (Fig S3. a–e). Hence, we sought to investigate whether FNDC5 deficiency induced post-translational modification of these proteins, and found that FNDC5 deficiency significantly enhanced the acetylation level of OPA1, while there is no obvious change of acetylation level in DRP1 (Fig. S3f). Notably, it has been reported that acetylation of OPA1 could significantly dampen its activity, while SIRT3 is the upstream deacetylase of OPA1, and is also acknowledged as the most abundant deacetylase and a critical regulatory center in mitochondria [33, 34]. We further found that FNDC5 knockout induced the downregulation of SIRT3 at both the transcriptional and protein levels (Fig. 4l–n). Isolation of the mitochondria and examination of acetylation levels showed increased acetylation of mitochondrial proteins in the FNDC5-knockout group (Fig. 4o, p), indicating that FNDC5 knockout resulted in reduced SIRT3 levels and function.

In the present study, we also revealed that acetylated SOD2 (an important antioxidant enzyme located in mitochondria) was significantly enhanced in FNDC5 knockout cells (Fig. S3g, h). However, FNDC5 knockout did not affect mitophagy with no significant change in autophagic flux (Fig. S4a, b) and protein levels of HSP60 and Timm50, which are usually degraded during mitophagy (Fig. S4c–e). Furthermore, we tested whether honokiol (HKL, a pharmacological agonist of SIRT3) could alleviate FNDC5 deficiency-induced damage to mitochondrial quality control. The experimental results suggested that activation of SIRT3 improved the mitochondrial dynamics and bioenergetics (Fig. 4c–k), indicating impaired mitochondrial function. These results indicated that activation of SIRT3 alleviates FNDC5 deficiency-induced disruption of mitochondrial dynamics and bioenergetics.

### Neuronal SIRT3 deficiency reduces FNDC5-mediated protection of mitochondria in mice

**Fig. 5:**
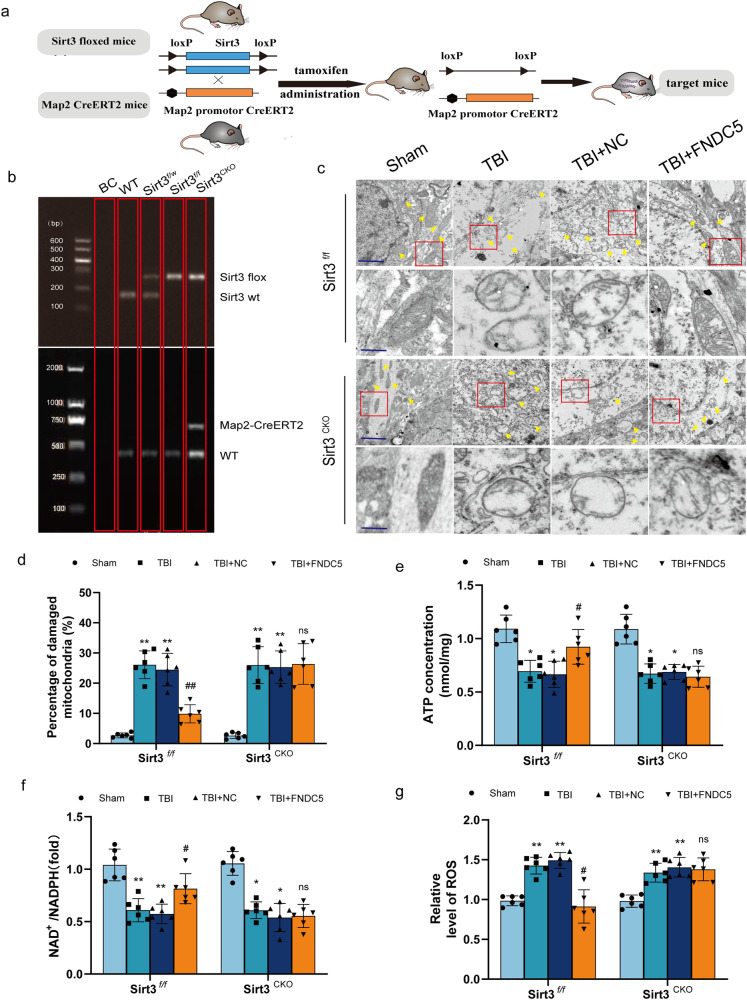
Neuronal SIRT3 deficiency reduces FNDC5-mediated protection of mitochondria in mice. **a** Experimental flowchart of neuronal-specific SIRT3 homozygous knockout mice (SIRT3 cKO). **b** Identification of neuronal-specific SIRT3 homozygous knockout mice (SIRT3 cKO). **c** Representative ultrastructure of neurons in each group and the magnified images of the ultrastructure of neurons shown in (**a**). Scale bar: 0.3 μm and 0.05 μm (*n* = 6 for each group). **d** Percentage of damaged mitochondria. **e** ATP content in brain tissue. **f** NAD^+^/NADH ratio in brain tissue. **g** Relative level of ROS in brain tissue, *n* = 6 for each group. Significance was determined by two-way repeated ANOVA (**d**–**g**) with Bonferroni post hoc tests. *P < 0.05 and **P < 0.01 vs. sham group, ^#^*P* < 0.05 and ^##^*P* < 0.01 vs. TBI + NC group. Values are presented as the mean ± SEM.

To validate the function of SIRT3 in FNDC5-mediated mitochondrial quality control, neuronal SIRT3 conditional knockout mice (SIRT3 cKO) were established (Fig. 5a, b). Electron microscopy showed the presence of normal mitochondria with elongated tubules and conspicuous cristae, together with several globular structures (Fig. 5c, d). After TBI, the mitochondria exhibited significant heterogeneity in both shape and size. The number of globular structures increased, indicative of fragmentation. Ruptured membranes, swelling, and distorted cristae were also visible. Overexpression of FNDC5 significantly improved these abnormalities. However, the protective effects of FNDC5 on mitochondrial morphology were diminished in SIRT3^cKO^ mice. Moreover, it was observed that the dysregulated mitochondrial bioenergetics (ATP production, the NAD + /NADH ratio and ROS level) were restored by FNDC5 overexpression. Consistently, the protective effects of FNDC5 on mitochondrial bioenergetics were counteracted in SIRT3^cKO^ mice (Fig. 5e–g).

### Neuronal SIRT3 knockout counteracts FNDC5-mediated effects on oxidative stress and neuronal apoptosis after TBI in mice

**Fig. 6:**
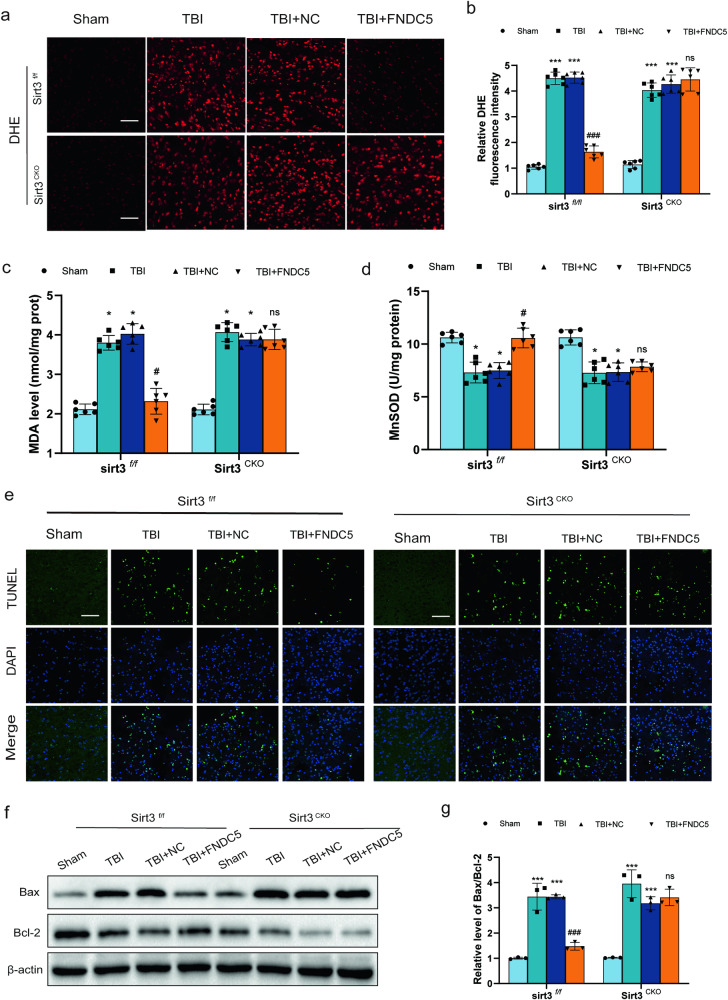
Neuronal SIRT3 knockout counters FNDC5-mediated effects on oxidative stress and neuronal apoptosis after TBI in mice. **a** Representative DHE staining images in SIRT3^CKO^ mice and SIRT3^f/f^ mice. Scale bar: 200 μm. **b** The quantitative results of DHE staining images. **c** The effects of FNDC5 on MDA levels in SIRT3^CKO^ mice and in SIRT3^f/f^ mice. **d** The effects of FNDC5 on mitochondrial MnSOD activity in SIRT3^CKO^ mice and in SIRT3^f/f^ mice. **e** Representative images of TUNEL staining of the perilesional cortex 24 h in SIRT3^CKO^ mice and SIRT3^f/f^ mice. Scale bar: 200 μm. **f**, **g** Representative Western blot and statistical analysis of the levels of Bax and Bcl-2 in SIRT3^CKO^ mice and in SIRT3^f/f^ mice. (*n* = 6 for each group). Significance was determined by two-way repeated ANOVA (**b**, **c**, **d**, **g**) with Bonferroni post hoc tests. *P < 0.05 and **P < 0.01 vs. sham group, ^#^*P* < 0.05 and ^##^*P* < 0.01 vs. TBI + NC group. Values are presented as the mean ± SEM.

We next investigated whether the FNDC5-mediated effects on oxidative stress and neuronal apoptosis depend on SIRT3. DHE staining found that FNDC5 could not significantly reduce TBI-induced ROS production (Fig. 6a, b), MDA levels (Fig. 6c) and MnSOD activity in the brain of SIRT3^cKO^ mice (Fig. 6d). In addition, TUNEL staining indicated that overexpression of FNDC5 mitigated neuronal apoptosis after TBI in SIRT3^f/f^ mice, but not in SIRT3^cKO^ mice (Fig. 6e). Western blot consistently showed that the ratio of Bax/Bcl-2 was decreased (Fig. 6f, g). Overall, these results indicated that neuronal SIRT3 knockout counteracted FNDC5-mediated effects on oxidative stress and apoptosis after TBI in mice.

### FNDC5 upregulates the level of SIRT3 by modulating the protein stability of NRF2

**Fig. 7:**
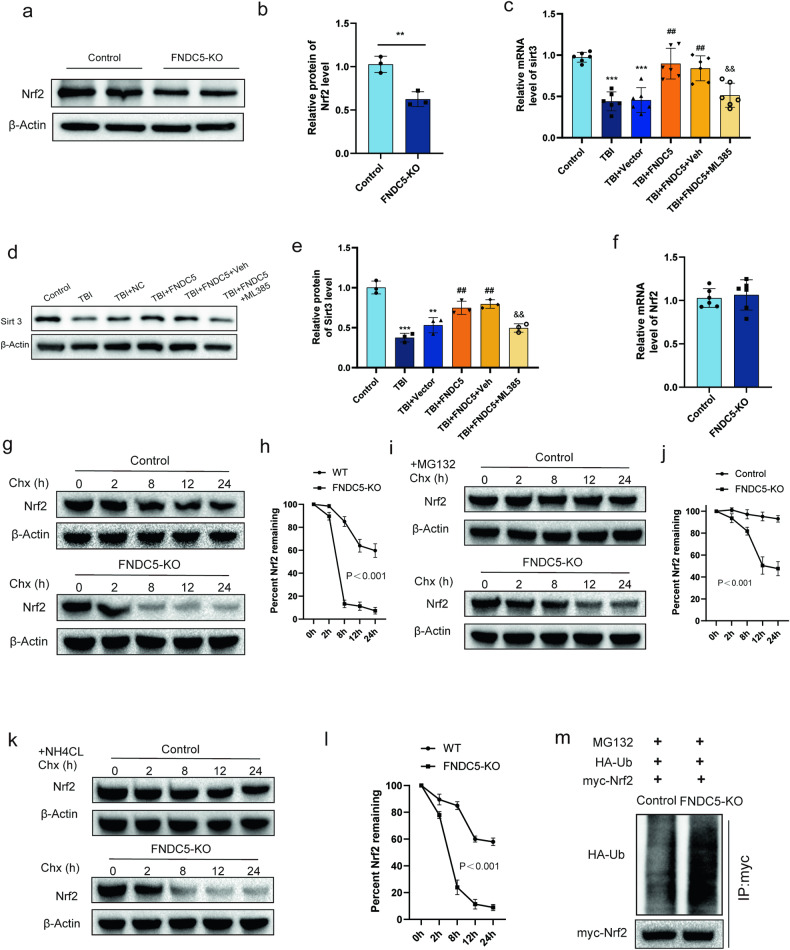
FNDC5 upregulates the level of SIRT3, and mechanically, through modulating the protein stability of NRF2. **a**, **b** Western blot and statistical analysis of the levels of NRF2 expression in normal HT22 cell line and FNDC5 KO HT22 cell line. **c** Relative mRNA level of SIRT3 after treating with an NRF2 inhibitor (ML385). **d**, **e** Western blot and statistical analysis of the levels of SIRT3 expression. **f** FNDC5 deficiency does not affect the mRNA of NRF2. **g** The protein level of NRF2 was degraded faster over time in FNDC5 knockout cells after incubating with the mRNA synthesis inhibitor CHX. **h** statistical analysis of the levels of NRF2. **i** The protein level of NRF2 expression after incubating with the proteasome inhibitor MG132. **j** Statistical analysis of the levels of NRF2. **k** The protein level of NRF2 expression after incubating with the lysosomal inhibitor NH4Cl. **l** statistical analysis of the levels of NRF2. **m** Ubiquitination level of NRF2. Significance was determined by Student’s *t* test (**b**, **f**) and one-way ANOVA (**c**, **e**) or two-way repeated ANOVA (**h**, **j**, **l**) with Bonferroni post hoc tests. *P < 0.05 and **P < 0.01 vs. Control group, ^#^*P* < 0.05 and ^##^*P* < 0.01 vs. TBI + vector group, & *P* < 0.05 and ^&&^*P* < 0.01 vs. TBI + FNDC5 + veh group. Values are presented as the mean ± SEM.

After establishing that FNDC5 affected the expression of SIRT3 from the transcriptional level, we hypothesized that FNDC5 might regulate the expression of SIRT3 through a transcription factor. NRF2 has been reported to bind directly to the promoter of SIRT3 and increase its expression. We found that the protein level of NRF2 was lower in the FNDC5 KO cell (Fig. 7a, b). Moreover, in vivo studies indicated that treatment with an NRF2 inhibitor (ML385) significantly counteracted the effect of FNDC5 on SIRT3 mRNA (Fig. 7c) and protein levels (Fig. 7d, e).

However, we found that FNDC5 deficiency does not affect the mRNA levels of NRF2 (Fig. 7f). After cycloheximide (CHX) was used to inhibit the mRNA synthesis of cells, we detected NRF2 protein levels. It was found that the protein was degraded faster over time in FNDC5 knockout cells (Fig. 7g, h), which suggested that FNDC5 deficiency may accelerate the degradation of NRF2 protein. Protease degradation occurs through two main pathways: ubiquitin-proteasome pathway and autophagy-lysosome pathway. To determine whether NRF2 is degraded via the lysosomal system or through the ubiquitin-proteasome system, the effect of the well-known lysosomal inhibitor NH4Cl or the proteasome inhibitor MG132 on the degradation of NRF2 was evaluated. The results showed that MG132 reversed the degradation of NRF2 in the absence of FNDC5 (Fig. 7i, j), while NH4Cl yielded no significant effect (Fig. 7k, l). Furthermore, we found that the ubiquitination level was higher after knocking out FNDC5 (Fig. 7m). These results indicated that FNDC5 regulates the expression of SIRT3 by modulating the protein stability of transcription factors NRF2.

### Nanoparticle-mediated irisin delivery to the brain significantly promotes recovery after TBI in mice

**Fig. 8:**
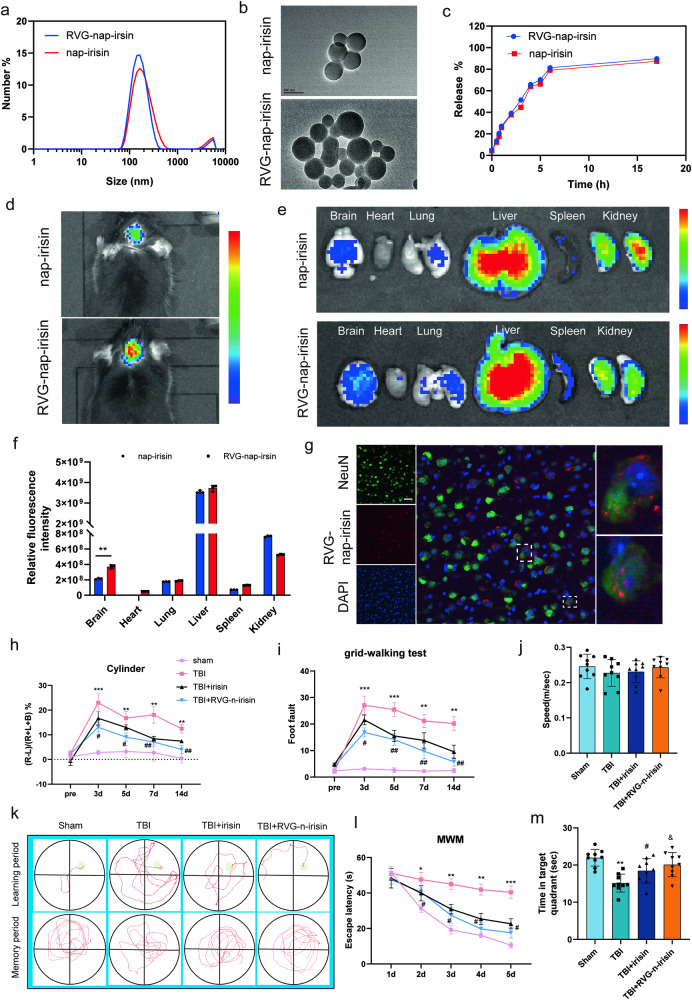
Nanoparticle-mediated irisin delivery to the brain significantly promotes recovery after TBI in mice. **a** TEM observation of nap-irisin and RVG-nap-irisin. **b** Particle size analysis of nap-irisin and RVG-nap-irisin. **c** The drug release kinetics of nap-irisin and RVG-nap-irisin. **d**–**f** Real-time fluorescence imaging, and corresponding fluorescence analysis of mice after intravenous injection of Cy5.5-labeled nap-irisin and RVG-Nap-irisin. **g** Immunofluorescence of RVG-nap-irisin. **h**–**m** several behavioral tests with treatment with irisin or RVG-nap-irisin after TBI (*n* = 9 for each group). Significance was determined by one-way ANOVA (**j**, **m**) or two-way repeated ANOVA (**f**, **h**, **i**, **l**) with Bonferroni post hoc tests. *P < 0.05 and **P < 0.01 vs. sham group, ^#^*P* < 0.05 and ^##^*P* < 0.01 vs. TBI + irisin group. Values are presented as the mean ± SEM.

Although the lentiviral targeting of FNDC5 is effective in experimental studies, the procedure is difficult to apply in clinical situations due to safety concerns and cost. Accordingly, we explored whether supplementation with irisin could be an efficient pharmacological solution for acute brain injuries. However, it is well established that the blood-brain barrier (BBB) may limit the efficacy of drugs delivered to the central nervous system to a large extent. To enhance the delivery efficiency, engineered RVG29-conjugated nanoparticles were generated (RVG-nap-irisin). As shown in Fig. 8a, b, the nanoparticles exhibited a uniform shape with a size below 200 nm. Figure 8c shows the drug release behavior of RVG-nap-irisin. Fluorescence imaging showed that RVG-nap-irisin could effectively pass through the BBB and enter the brain (Fig. 8d–f). Immunofluorescence showed that RVG-nap-irisin could be absorbed by mouse brain neurons (Fig. 8g).

Behavioral assessments showed that treatment with irisin or RVG-nap-irisin was effective for TBI, and RVG-nap-irisin presented a better therapeutic effect. As shown in Fig. 8h, i, mice administered RVG-nap-irisin showed a decreased proportion of foot faults in the grid-walking test and improved performance with decreased forelimb asymmetry in the cylinder test after TBI. Similar results were recorded during the MWM test (Fig. 8j–m), where TBI mice injected with RVG-nap-irisin showed a shorter escape latency and longer time in the quadrant. These results indicated nanoparticle-mediated irisin delivery to the brain significantly promotes recovery after TBI in mice.

## Discussion

In recent years, the number of TBI cases has escalated worldwide. Unfortunately, the prognosis of TBI is often dismal due to its complex mechanism of occurrence and development. FNDC5/irisin is a myokine induced by physical exercise. There is a rich literature available substantiating that irisin is active in the central nervous system [18, 35]. However, the role of FNDC5/irisin in the perilesional tissue of the brain and secondary injury after TBI remains unclear. In the present study, the serum irisin concentrations were significantly higher in TBI patients with favorable outcomes and downregulated FNDC5 expression aggravated neurological deficits after TBI. Overexpression of FNDC5 could suppress oxidative stress, reduce neural apoptosis and improve neurological deficits after TBI. The activation of SIRT3 alleviated FNDC5 deficiency-induced damage to mitochondrial dynamics and bioenergetics. FNDC5 upregulated SIRT3 by modulating the protein stability of NRF2. Overall, the present study’s findings lay the groundwork for uncovering novel targets for neuroprotective treatment after TBI.

TBI often leads to vascular leakages, hemorrhage, hypoxia, and edema, and further pathological mechanisms include cell death within the meninges and brain parenchyma [7]. Following TBI, patients commonly suffer neurological deficits, cognitive decline, and behavioral alterations [36]. FNDC5/irisin is reportedly indispensable for neuronal development [17] and could rescue synaptic plasticity and memory defects of AD patients [18]. In our study, the serum levels of irisin in patients at 6 months post-TBI correlated with long-term outcomes. Furthermore, animal behavior experiments substantiated that overexpression of FNDC5 reduced lesion volume, attenuated brain edema and improved neurological function after TBI, indicating its possible neuroprotective activity.

Growing evidence suggests that impairment of endogenous antioxidant defense mechanisms results in oxidative stress, which plays a vital role in secondary events after TBI, often leading to neuronal death [37, 38]. Moreover, overexpression of FNDC5 could attenuate oxidative damage and cell apoptosis, which have been documented in disease models of vascular aging, myocardial infarction, kidney injury, osteoarthritis and liver ischemia [24, 29, 30, 39, 40]. Guo et al. focused on the effect of FNDC5 on the oxidative stress of the blood–brain barrier after TBI [15]. In this article, we focused on the effect of oxidative stress on the secondary injury of brain parenchyma after TBI and discussed its mechanism. Consistently, we observed the benefits of FNDC5 on oxidative stress and neuronal apoptosis. Then animal behavior experiments further validated the unique ability to promote brain recovery in mice after TBI.

Mitochondrial dysfunction leads to the generation of free radicals following apoptosis after TBI [36]. Overwhelming evidence substantiates that irisin could inhibit apoptosis and increase mitochondrial content in cells by decreasing excessive mitochondrial fission [29, 30]. To study the biological effect of FNDC5, an FNDC5 KO cell line was generated. We found that FNDC5 deficiency at baseline elicited mitochondrial morphology and mitochondrial biogenesis. Then, we investigated the mechanisms underlying alleviated mitochondrial damage associated with FNDC5 deficiency.

Then, we sought to investigate whether the key proteins regulating mitochondrial dynamics (DRP1, MFN1, MFN2 and OPA1) play a vital role in this process. No significant change in their protein expression levels was found, which may be attributed to the fact that post-translational modifications of mitochondrial dynamic proteins are not affected by the effect of FNDC5 on mitochondrial function. An increasing body of evidence suggests that acetylation of OPA1 could be induced by pathological stress, which affects mitochondrial metabolism [33, 34, 41]. In our study, FNDC5 deficiency significantly enhanced the acetylation level of OPA1, firstly unraveled the unique role of OPA1 underlying the FNDC5’s regulatory role on the mitochondrial dynamics.

Current evidence further indicates that upstream deacetylase of OPA1, e.g., SIRT3, an important deacetylase mainly within mitohondria, plays a critical role in modulating mitochondrial dynamics, ROS generation and ATP synthesis, which was similar to the previous reports [42]. Of note, we found that FNDC5 knockout induced the downregulation of SIRT3 at the transcriptional and protein levels. We further found that FNDC5 deficiency dysregulated the mitochondrial dynamics and bioenergetics, which was reversed by SIRT3 activation. SOD2 has been reported to protect cells from oxidative damage by catalyzing the dismutation of superoxide radicals into hydrogen peroxide and oxygen, and SIRT3 effectively promoted its activity through lysine deacetylation [43**–**45]. In our study, WB analysis revealed that acetylated SOD2 was significantly enhanced in FNDC5 knockout cells. Then, the pharmacological agonist of SIRT3 HKL was used. We found that activation of SIRT3 improved the mitochondrial dynamics and alleviated FNDC5 deficiency-induced damage to mitochondrial quality control. The above results suggested that FNDC5 deficiency led to dysregulated mitochondrial dynamics and bioenergetics, which was reversed by activation of SIRT3, which contributed to the hyperacetylation and inactivation of key regulatory proteins of mitochondrial dynamics and function, like OPA1 and SOD2. SIRT3^cKO^ mice were established, and experiments verified that the effects of FNDC5 on oxidative stress, neuronal apoptosis and mitochondrial bioenergetics were mediated by SIRT3. And the present study firstly reported that Neuronal SIRT3 knockout counteracts FNDC5-mediated effects on oxidative stress and neuronal apoptosis after TBI in mice, unravel the mechanism and the crucial factors for the FNDC5’s regulatory role on the mitochondrial dynamics and bioenergetics after TBI.

Furthermore, the mechanism of favorable effects of FNDC5 against oxidative stress and mitochondrial dysfunction after TBI by SIRT3 activation remains unclear. Given that FNDC5 knockout could induce the downregulation of SIRT3 at the transcriptional level, we assumed that FNDC5 might regulate the expression of SIRT3 through a transcription factor. NRF2 has been reported to be a novel regulator of SIRT3, which directly binds to the promoter of SIRT3 and increases its expression [46, 47]. In the present study, we found that FNDC5 knockout reduced the protein level of NRF2 but did not affect the mRNA of NRF2. The following experiments demonstrated that FNDC5 deficiency might accelerate the degradation of NRF2 protein through the ubiquitin-proteasome pathway. We first demonstrated that FNDC5 might affect the stability of NRF2 protein through the ubiquitin-proteasome pathway to regulate the expression of SIRT3, which plays a vital role in oxidative stress and mitochondrial dysfunction after TBI.

Guo et al. found that FNDC5/Irisin could alleviate BBB damage and inhibit oxidative stress after TBI, and the effect of irisin on the BBB after TBI could be alleviated by promoting mitochondrial UCP2 expression and may inhibit oxidative stress [15]. Herein, we confirmed that FNDC5 relieve the perilesional tissue injury after TBI by regulating the oxidative stress and mitochondrial dysfunction, which was depending on the change of SIRT3 transcriptional expression through modulating the protein stability of transcription factors NRF2. This mechanism was totally different from the FNDC5’s role to alleviating the BBB damage after TBI in previous report. These findings firstly inferred that FNDC5 might exhibit protective effects on different brain structures during TBI via specific meachnism and pathway.

Although the lentivirus used for targeting FNDC5 can be used for experiments, it is not practical for clinical applications, emphasizing the need for an efficient pharmacological solution. The BBB is a significant obstacle to drug delivery to the brain. NPs are efficient carriers for targeted drug delivery and sustained-release drug delivery. Surface modification of nanoparticles, such as coupling RVG polypeptide, can enhance penetration of the BBB and target the brain. Nonviral gene transfection with nanoparticles is an effective way to deliver drugs. For clinical translation, we constructed an engineered RVG29-conjugated nanoparticle system to deliver irisin to the brain and confirmed its promising therapeutic effects in a TBI mice model, with significant alleviated neurological deficits observed. These findings suggest that FNDC5/irisin may effectively treat acute brain injury.

The above studies overlap in their assertion that FNDC5/irisin may exert multiple positive effects on metabolic or nonmetabolic diseases, including brain health. Importantly, we found FNDC5/irisin may be a promising therapeutic target for acute brain injury in a mouse model of TBI and significant heterogeneity surrounds the pathophysiological factors of TBI patients, including the injury position, severity, and duration. None of the TBI mouse models currently in use can completely mimic the TBI injury disease process. Hence, further investigation into clinical applications using various animal models, larger samples and different techniques is warranted. This approach could potentially be used for clinical treatment. Nonetheless, more animal experiments and clinical trials are needed to prove its effectiveness.

In conclusion, the effective role of FNDC5/irisin in the perilesional tissue of the brain and secondary injury after TBI was verified in this paper. Our findings suggest that FNDC5/irisin has potential for treating TBI by enhancing SIRT3-mediated mitochondrial quality control.

### Supplementary information

Supplementary_materials

Original Data File

## Data Availability

The raw data supporting the conclusions of this article will be made available by the authors, without undue reservation.
