# Correction: FNDC5 prevents oxidative stress and neuronal apoptosis after traumatic brain injury through SIRT3-dependent regulation of mitochondrial quality control

**DOI:** 10.1038/s41419-024-06901-5

**Published:** 2024-07-24

**Authors:** Yufeng Ge, Xun Wu, Yaning Cai, Qing Hu, Jin Wang, Shenghao Zhang, Baocheng Zhao, Wenxing Cui, Yang Wu, Qiang Wang, Tian Feng, Haixiao Liu, Yan Qu, Shunnan Ge

**Affiliations:** 1 Department of Neurosurgery, Tangdu Hospital, Fourth Military Medical University, Xi’an, 710038 Shaanxi China; 2 Department of Ambulant Clinic, Political Work Department of People’s Republic of China Central Military Commission, Beijing, China

**Keywords:** Post-traumatic stress disorder, Brain injuries

Correction to: *Cell Death and Disease* 10.1038/s41419-024-06748-w, published online 27 May 2024

In this article typos have been corrected:

1. Consistently, the protective effects of FNDC5 on mitochondrial bioenergetics were counteracted in SIRT3 cKO mice.
2. Figure legends: Fig. 1: a, Serum irisin levels were decreased in patients with poor outcomes compared to those with good outcomes (*P* < 0.001).
3. The sentence ‘while there is no obvious change of acetylation level in DRP1’ has been deleted.
4. Fig. 2: the MRI scan of the brain in the bottom panels of Fig. 2b were incorrect due to the mistaken images being inadvertently inserted during the assembly of Fig. 2 (separate attachments in e-mail named original Fig. 2 and amended Fig. 2).

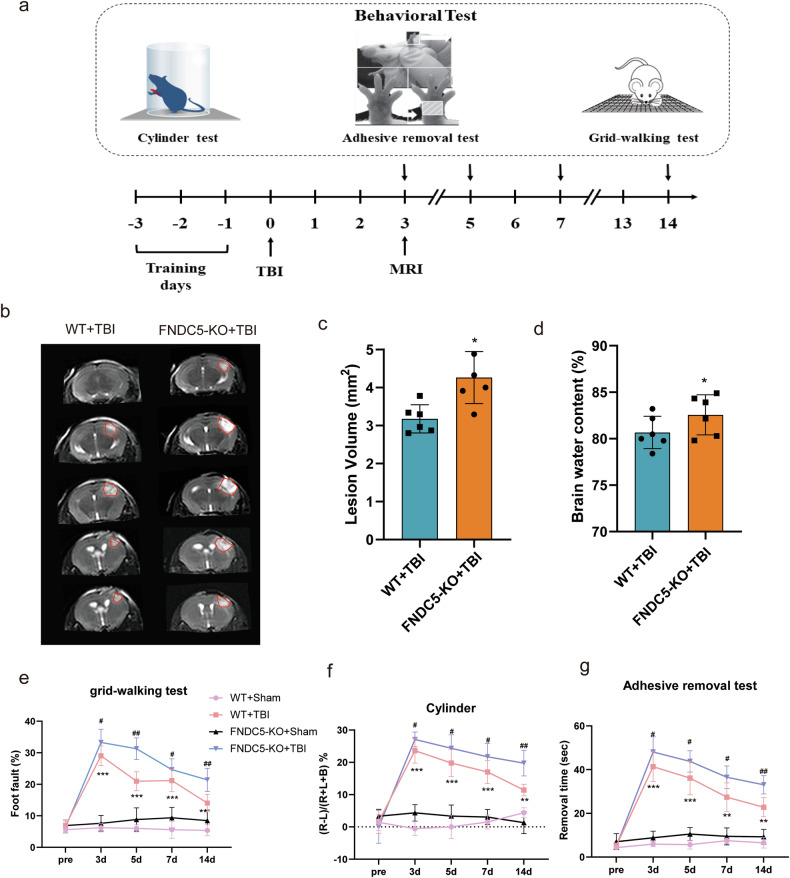
 
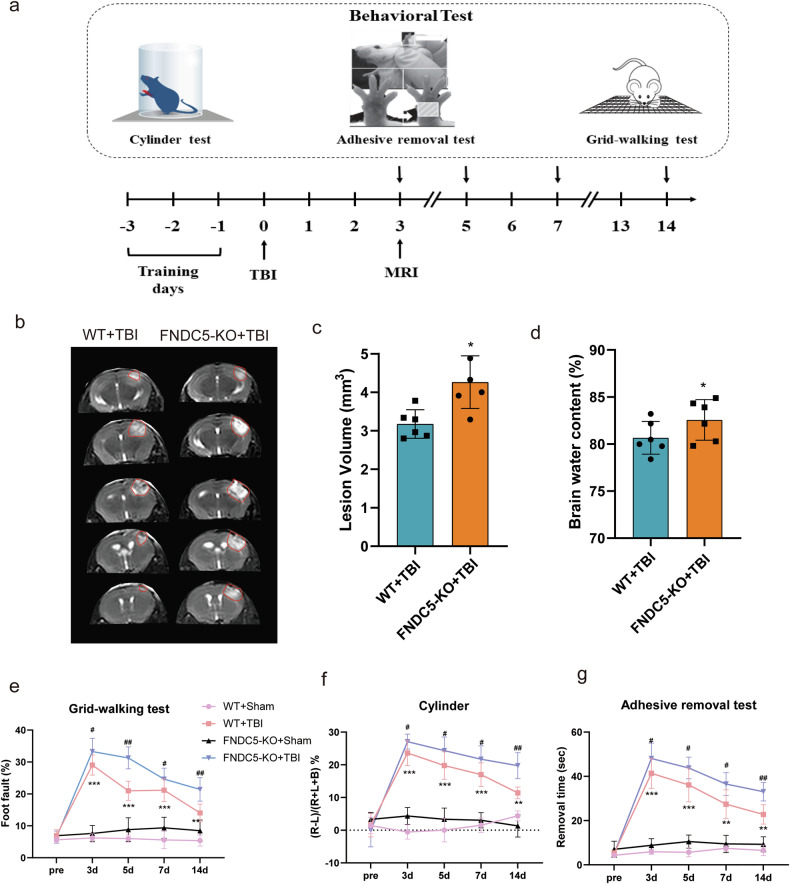


The original article has been corrected.

