# Supplementary material for: FNDC5 prevents oxidative stress and neuronal apoptosis after traumatic brain injury through SIRT3-dependent regulation of mitochondrial quality control
Source: Cell Death Dis. 2024 May 27;15(5):364. doi: 10.1038/s41419-024-06748-w (PMC11130144; doi:10.1038/s41419-024-06748-w)
Supplement: Supplementary file 1 — Supplementary_materials [file 41419_2024_6748_MOESM1_ESM.pdf]

Supplementary Materials for

FNDC5 prevents oxidative stress and neuronal apoptosis after traumatic brain injury through SIRT3-dependent regulation of mitochondrial quality control

Yufeng Ge[1†], Xun Wu[1†], Yaning Cai[1†], Qing Hu[1], Jin Wang[1], Shenghao Zhang[1], Baocheng Zhao[2], Wenxing Cui[1], Yang Wu[1], Qiang Wang[1], Tian Feng[1], Haixiao Liu[1], Yan Qu [1,*], and Shunnan Ge [1,*]

[1] Department of Neurosurgery, Tangdu Hospital, Fourth Military Medical University, Xi'an 710038, Shaanxi, China

[2] Department of Ambulant Clinic, Political Work Department of People's Republic of China Central Military Commission, Beijing, China.

† These authors contributed equally to this work.

[*] Corresponding author：

Yan Qu, MD, PhD, email: yanqu0123@fmmu.edu.cn.

Shunnan Ge, MD, PhD, email: gesn8561@fmmu.edu.cn.

This word file includes:

Supplementary Fig. S1 to S4, and figure legends

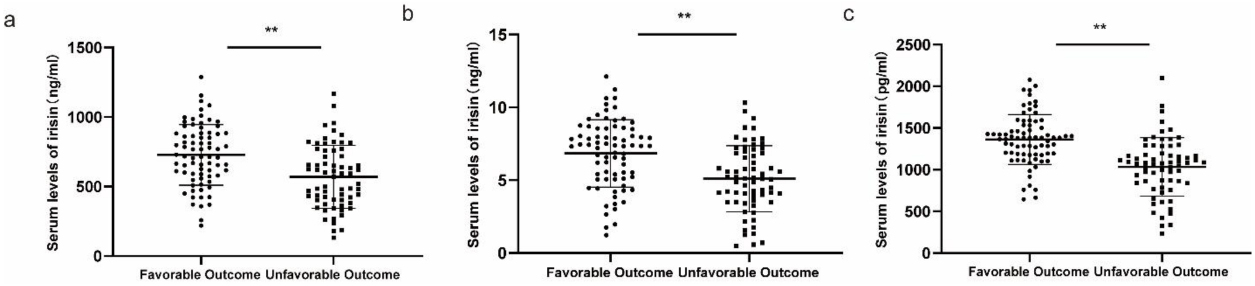

Fig. S1. Serum irisin levels of patients. **a,** Serum levels of irisin detect with commercial ELISA kit of RAG018R, BioVendor R&D. **b,** Serum levels of irisin detect with commercial ELISA kit of NBP3-08117, Novus. **c,** Serum levels of irisin detect with irisin-specific Luminex bead-based multiplex detection system (Merck Millipore). Significance was determined by Student's t-test (**a-c**). Values are presented as the mean ± SD.

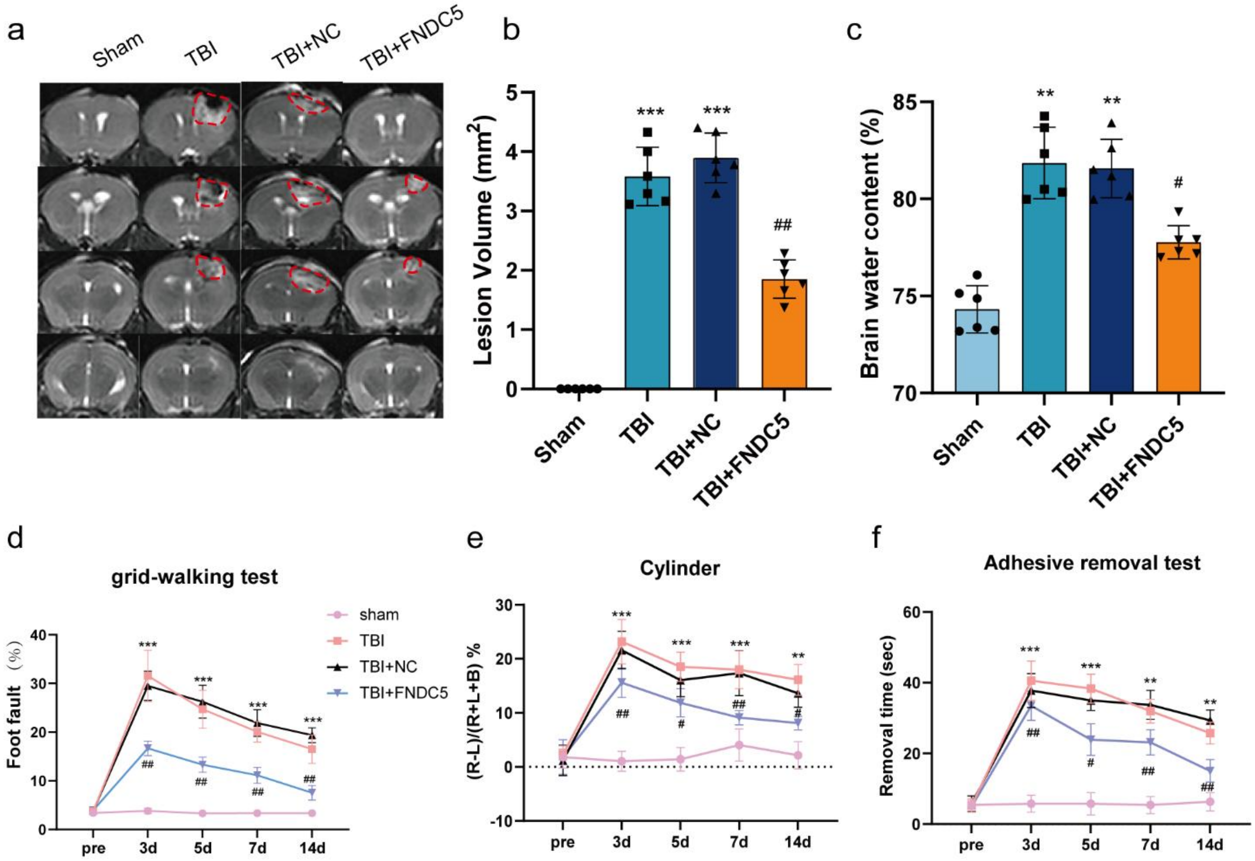

Fig. S2. Overexpression of FNDC5 reduces lesion volume, attenuates brain edema and improves neurological function after TBI. **a,** MRI scan of the brain in each group; n = 6 for each group. **b,** Quantification of lesion volume of MRI. **c,** measurement of brain water content. **d-f,** several behavioral tests after TBI (n = 9 for each group). Significance was determined by one-way ANOVA (**b, c**) or two-way repeated ANOVA (**d, e, f**) with

Bonferroni post hoc tests. *P <0.05 and **P <0.01 vs. Sham group, #P <0.05 and ##P <0.01 vs. TBI group. Values are presented as the mean ± SEM.

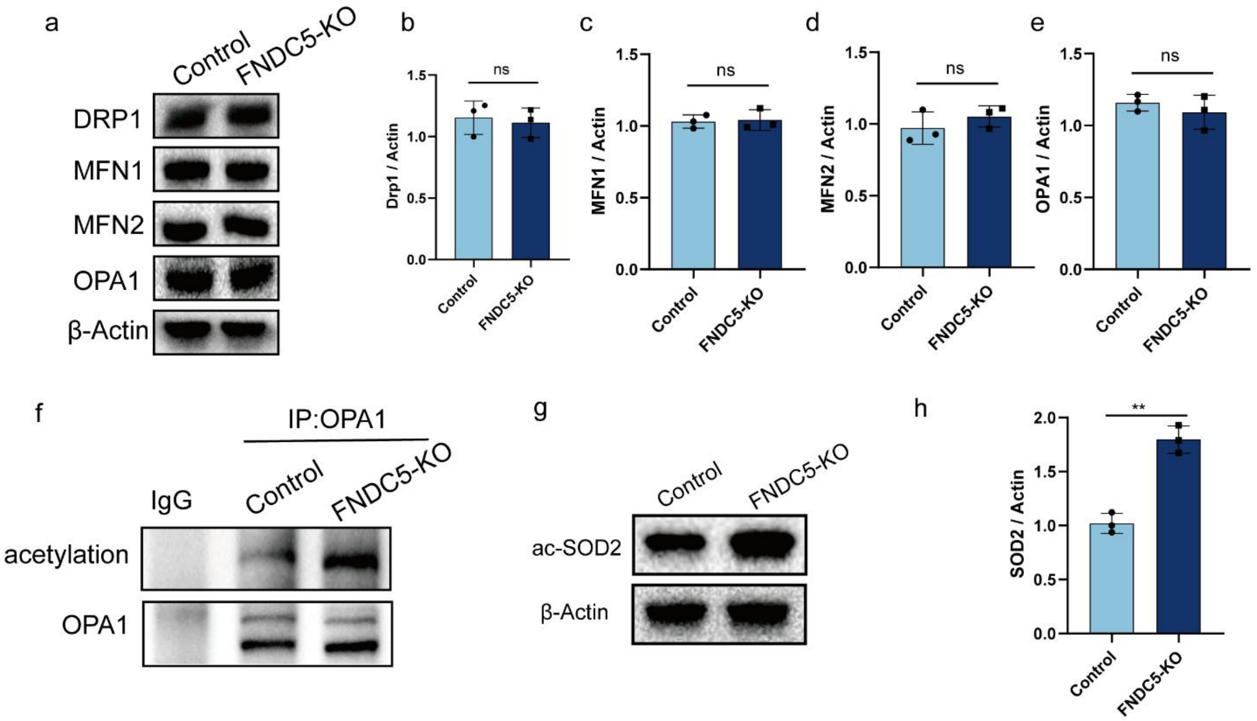

Fig. S3. FNDC5 deficiency did not change the protein expressions regulating mitochondrial dynamics. **a-e,** Western blots and statistical analysis of DRP1, MFN1, MFN2 and OPA1 expression. **f,** FNDC5 deficiency significantly enhanced the acetylation level of OPA1. **g-h,** acetylated SOD2 was significantly enhanced in FNDC5 knockout cells. Significance was determined by Student's t-test (**b-e, h**). *P <0.05 and **P <0.01 vs. Sham group, #P <0.05 and ##P <0.01 vs. TBI group. Values are presented as the mean ± SEM.

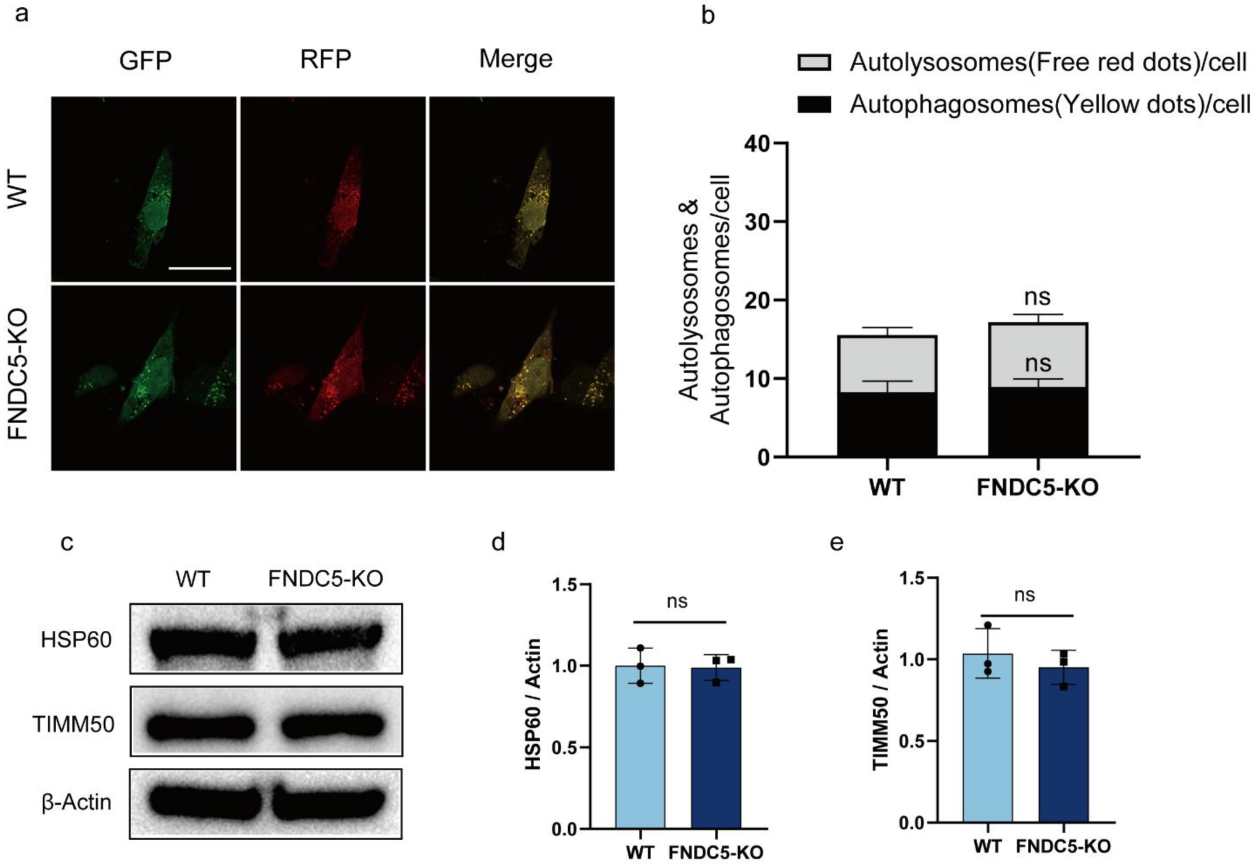

Fig. S4. FNDC5 knockout have no impact on mitophagy in the HT22 cell line. **a-b**, FNDC5 knockout have no impact on autophagic flux in the HT22 cell line. (n = 6 for each group). **c-e,** Western blots and statistical analysis of the levels of HSP60 and TIMM50 expression. Values are presented as the mean ± SD. Significance was determined by Student's t-test (**d, e**). *P <0.05 and **P <0.01 vs. WT group. Values are presented as the mean ± SEM.

Supplementary Material Table1

Glasgow Outcome Scale (GOS)	1	2	3	4	5
Number of patients (in this paper)	18	12	38	35	40
Description of GOS	Dead	Vegetative state	Severe disability (conscious but disabled)	Moderate disability (disabled but independent)	Good recovery (excellent recovery with a return to baseline functional status)
outcomes	unfavorable outcomes			favorable outcomes	
