# Supplementary material for: FNDC5 prevents oxidative stress and neuronal apoptosis after traumatic brain injury through SIRT3-dependent regulation of mitochondrial quality control
Source: Cell Death Dis. 2024 May 27;15(5):364. doi: 10.1038/s41419-024-06748-w (PMC11130144; doi:10.1038/s41419-024-06748-w)

Full Western Blots for Fig 3g

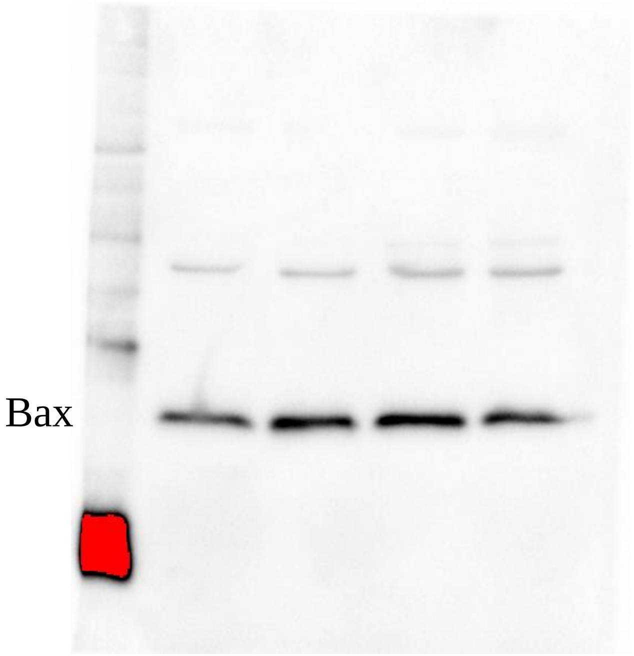

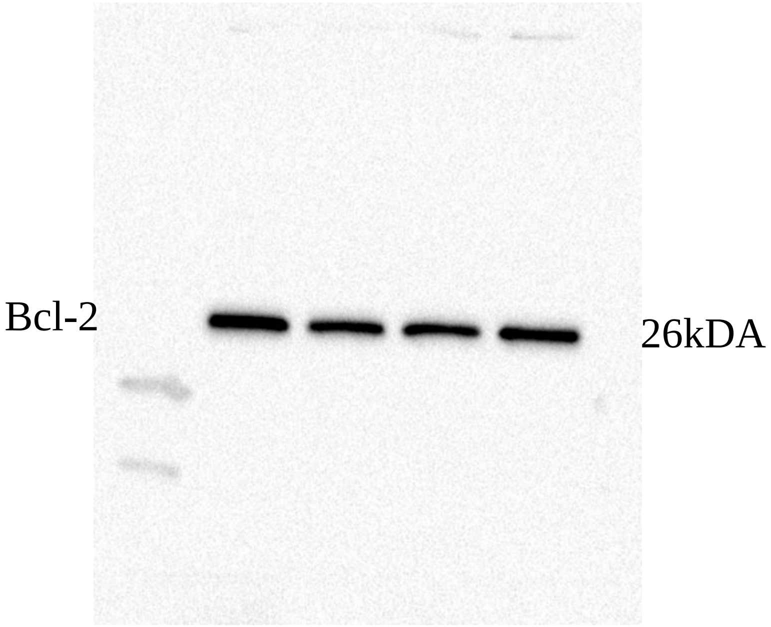

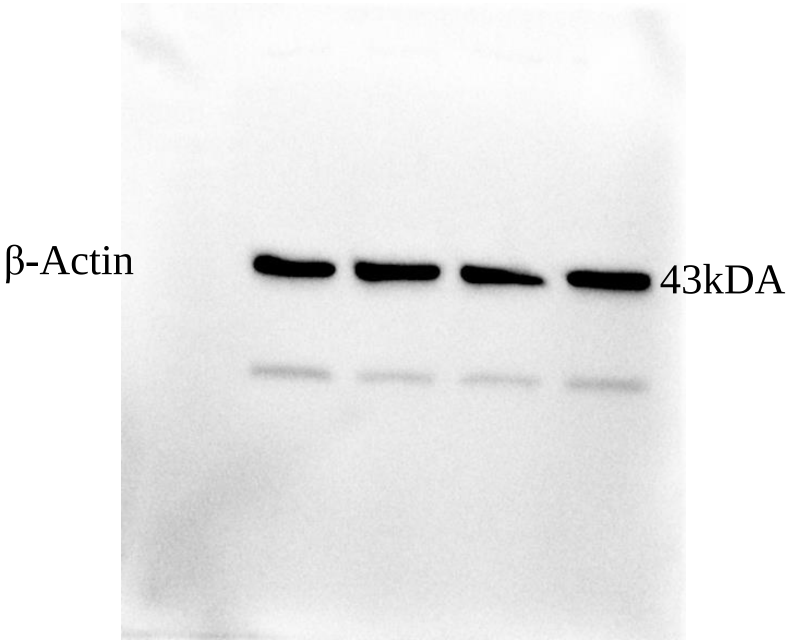

Full Western Blots for Fig 3a and 3l

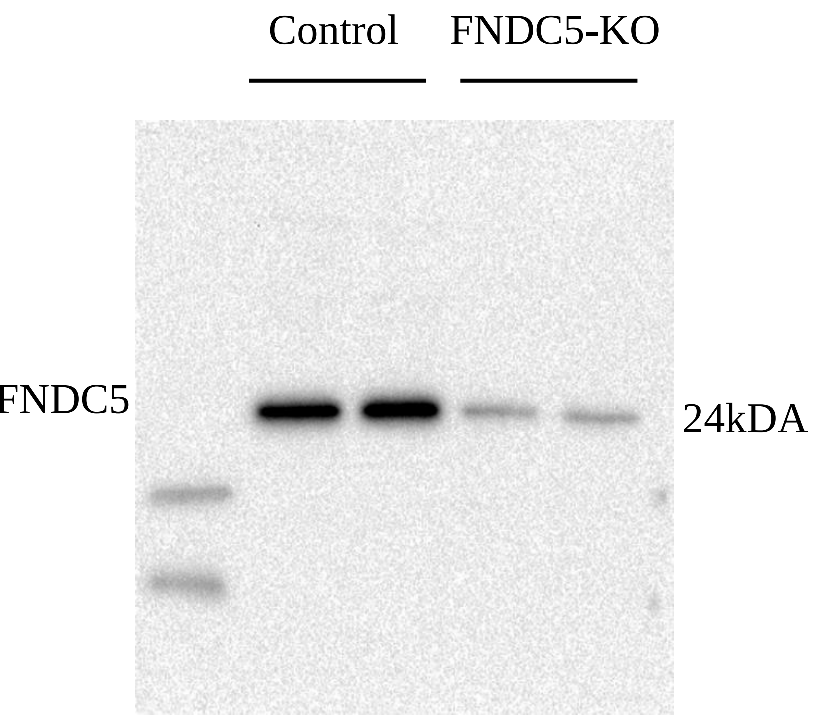

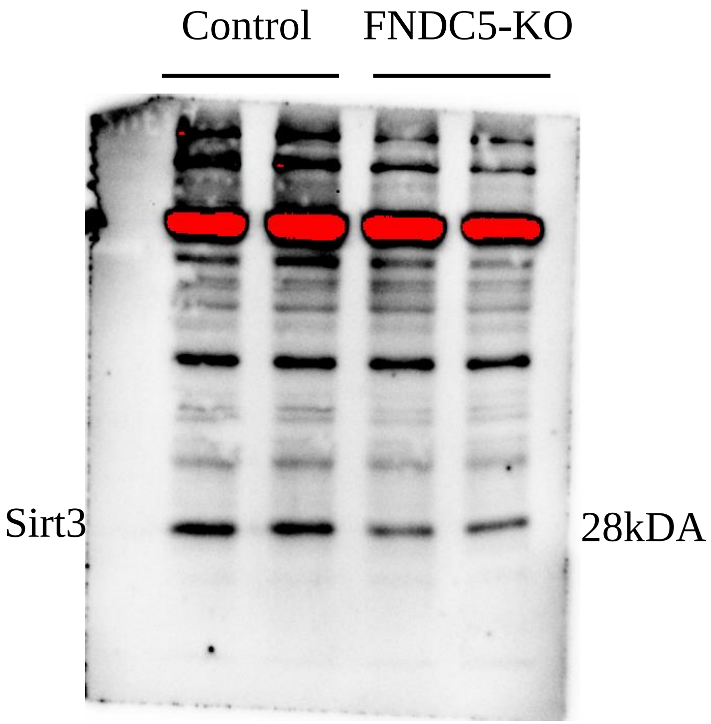

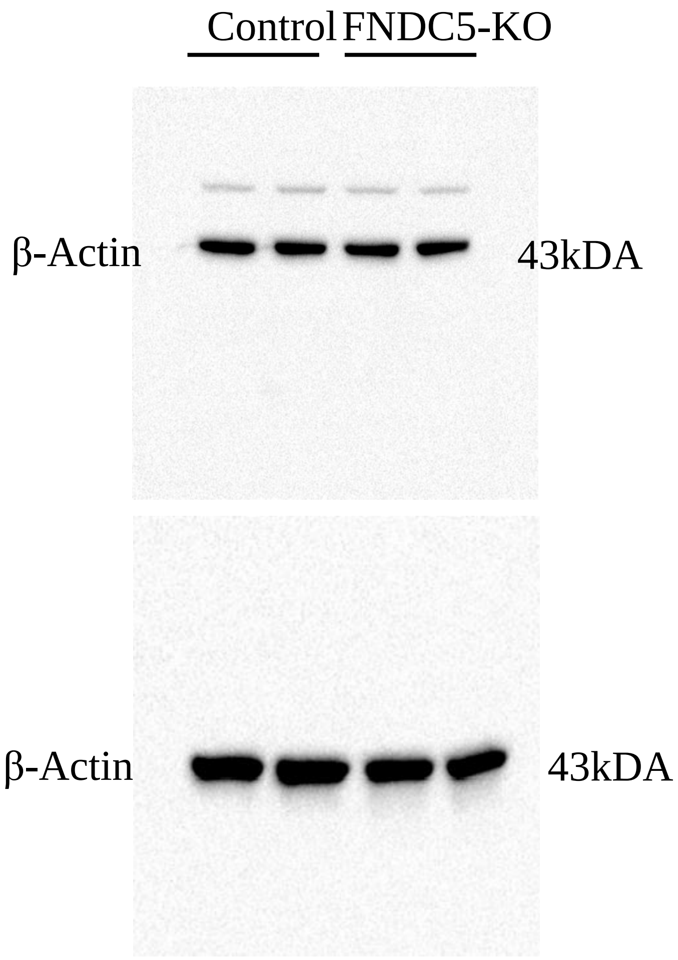

Full Western Blots for Fig 3o

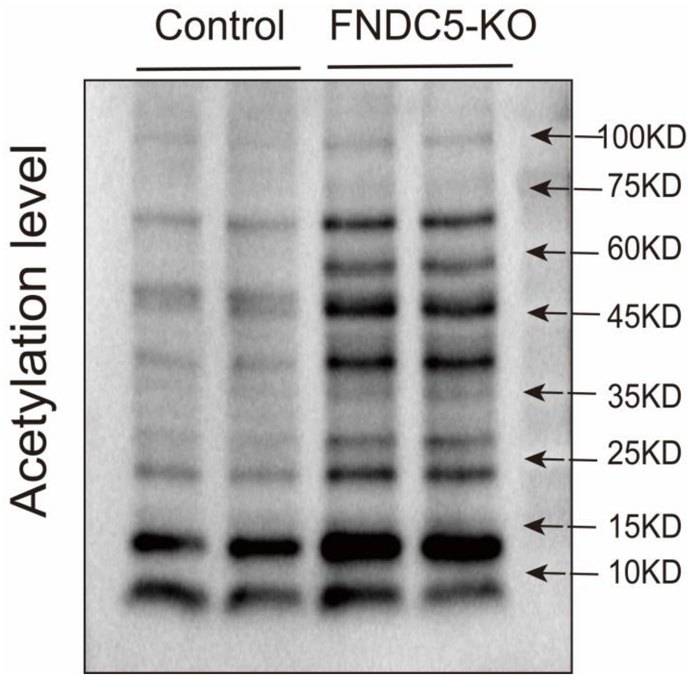

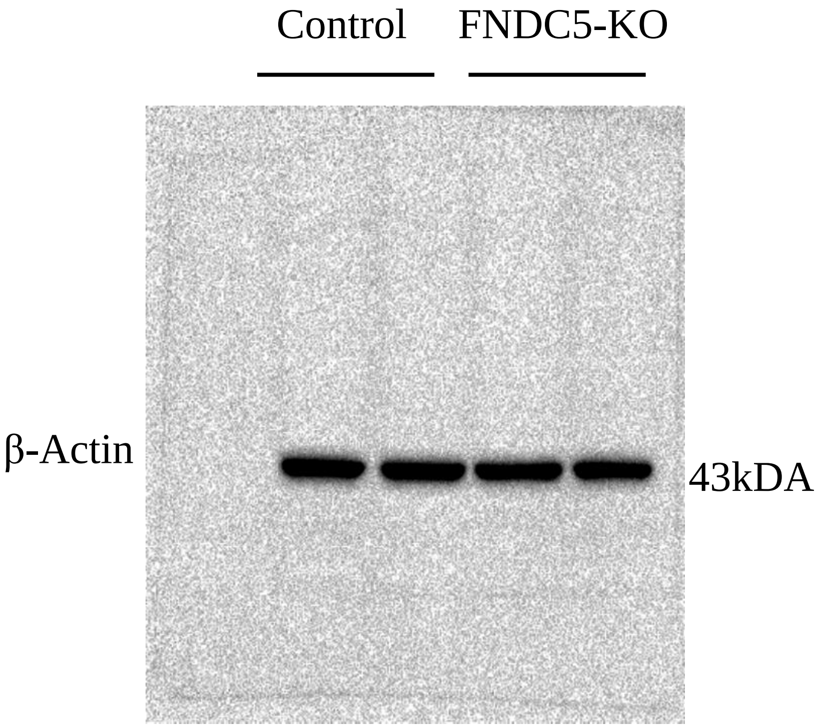

Full Western Blots for Fig 6f

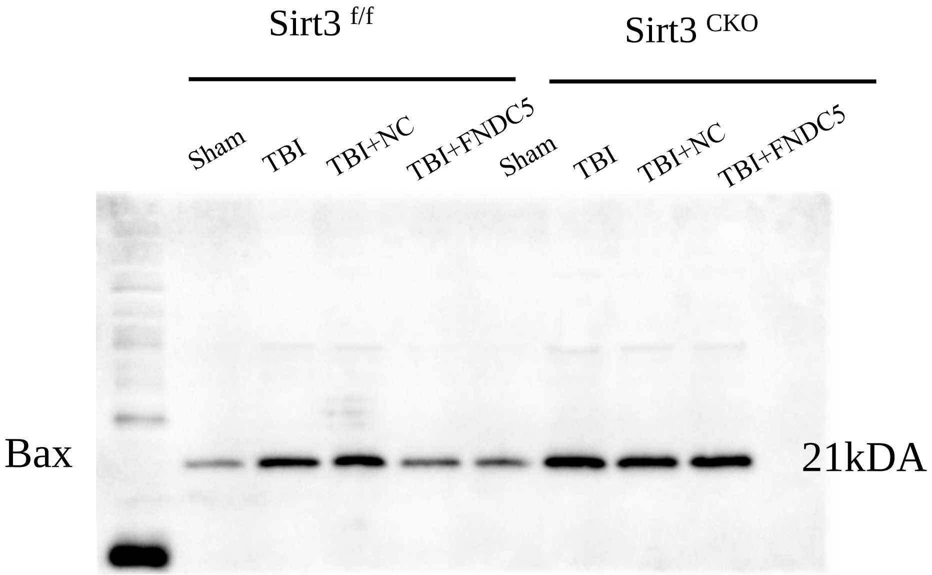

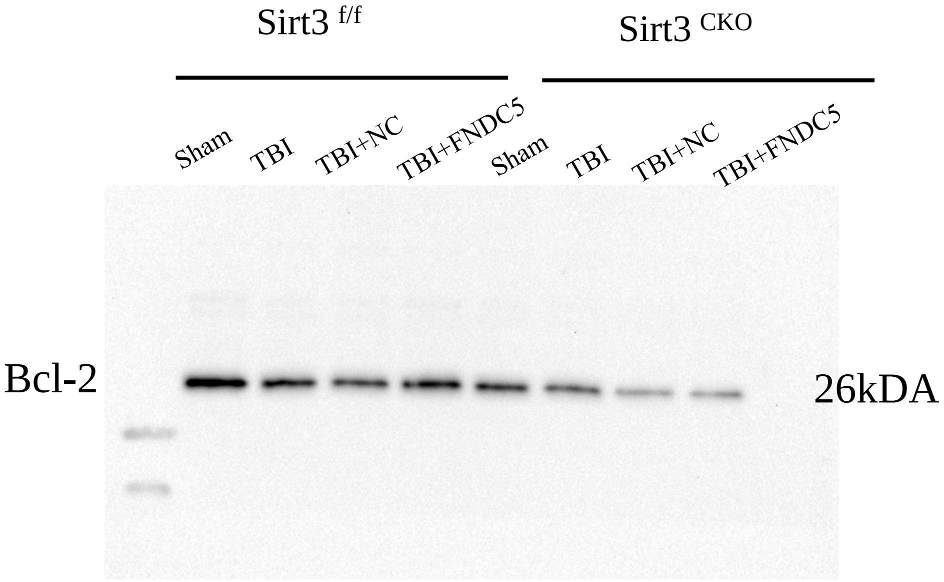

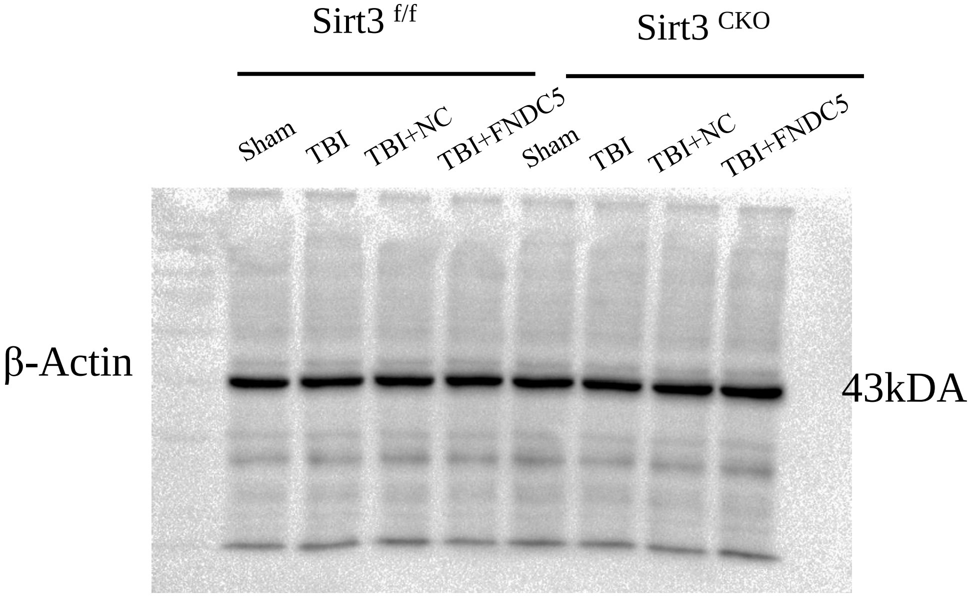

Full Western Blots for Fig 7a

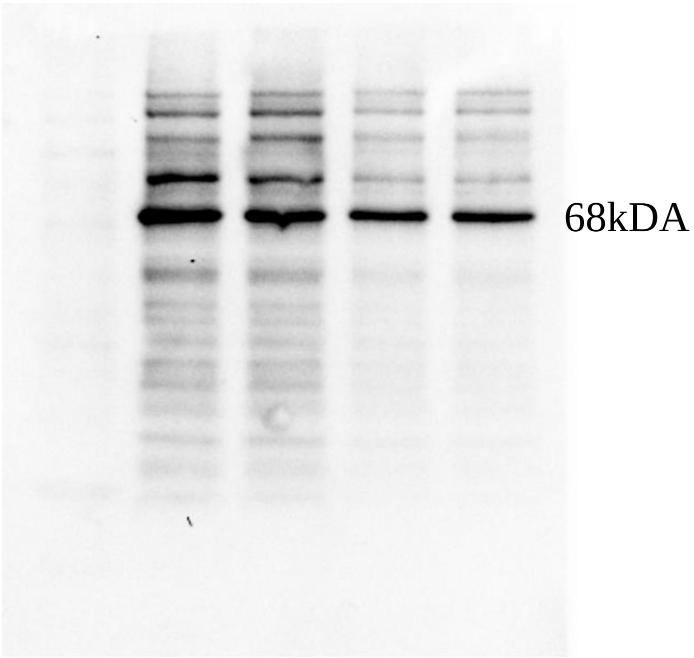

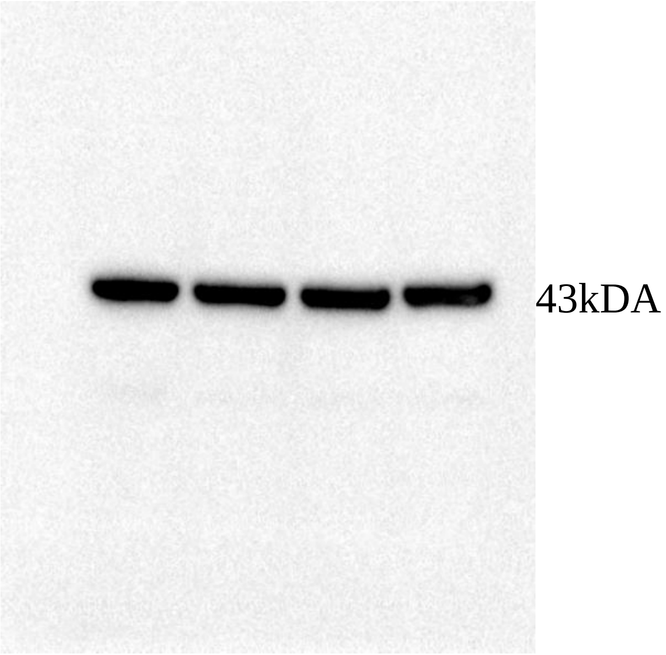

Full Western Blots for Fig 7d

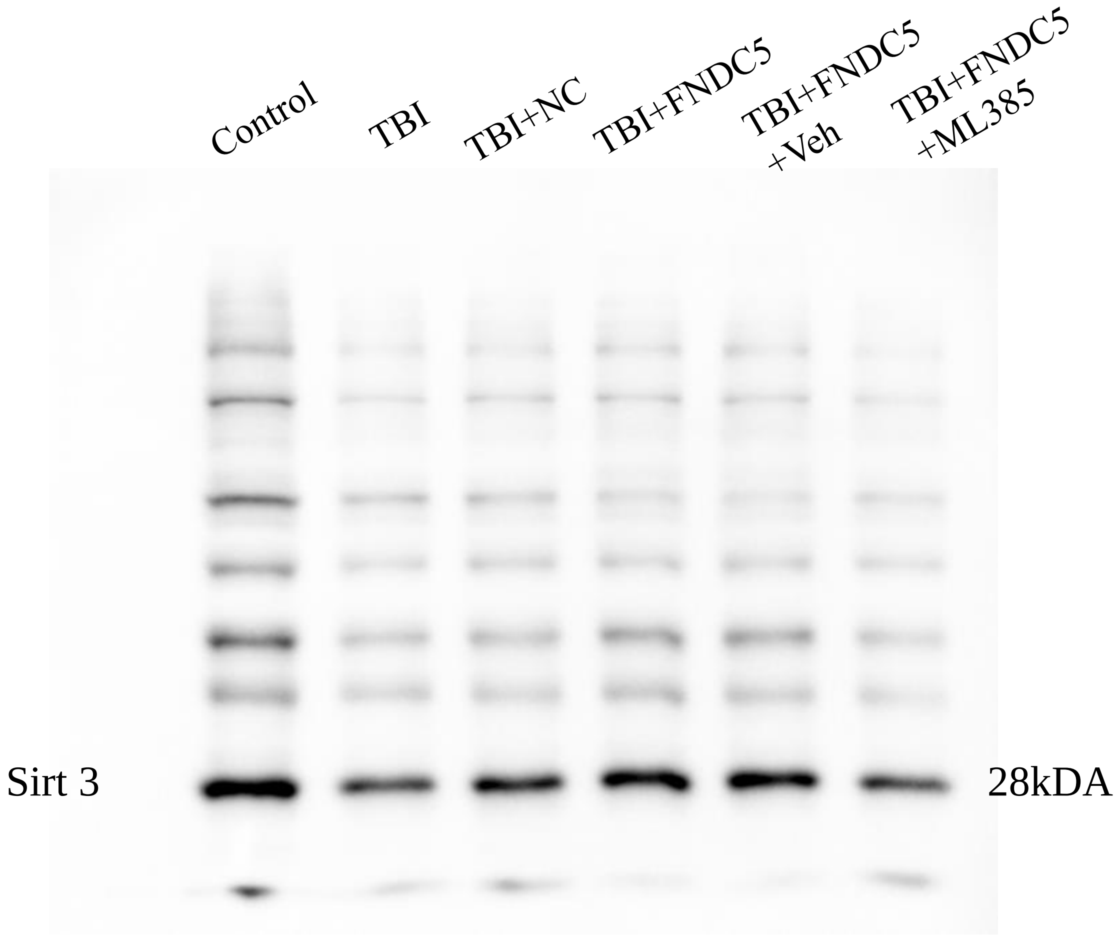

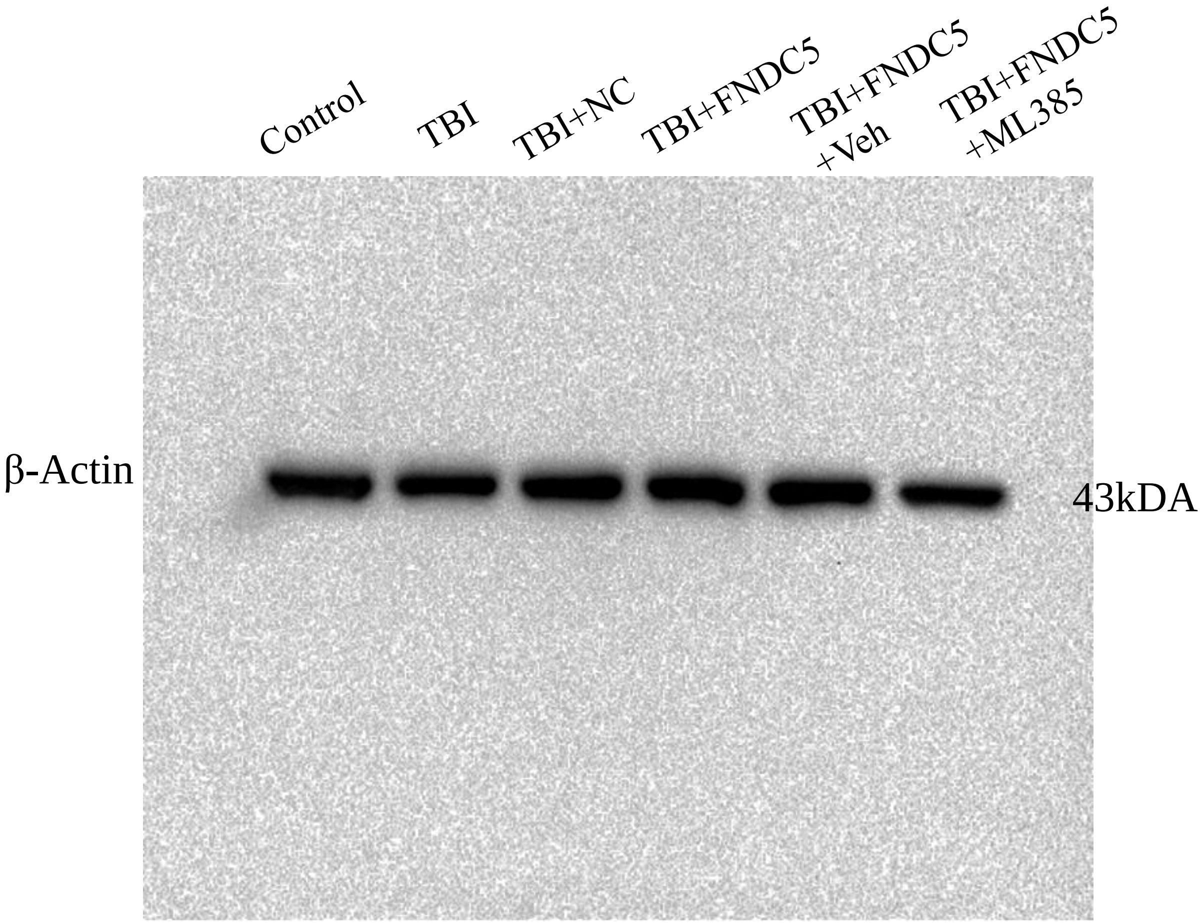

Full Western Blots for Fig 7g

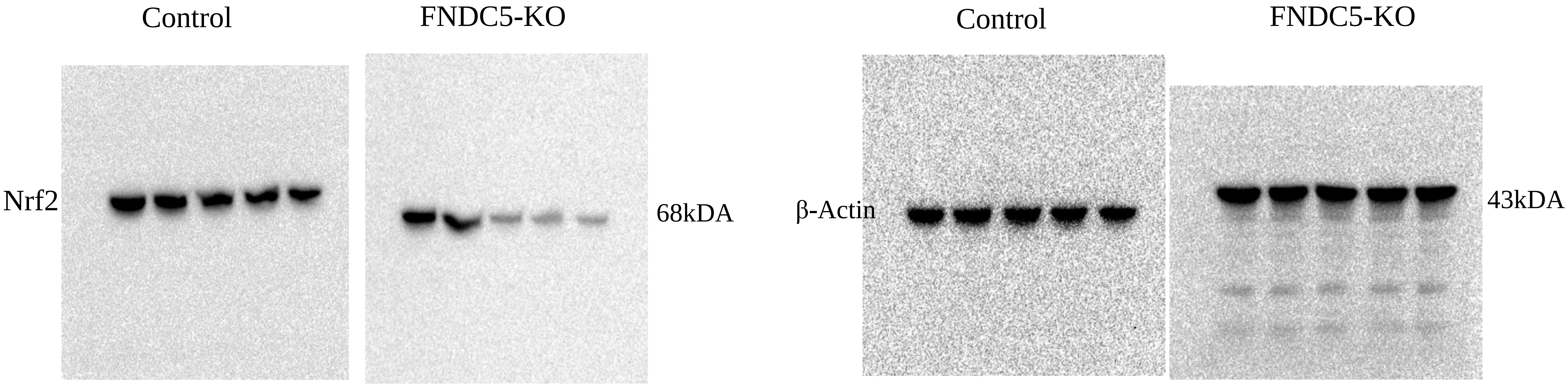

Full Western Blots for Fig 7i

Control FNDC5-KO

Nrf2

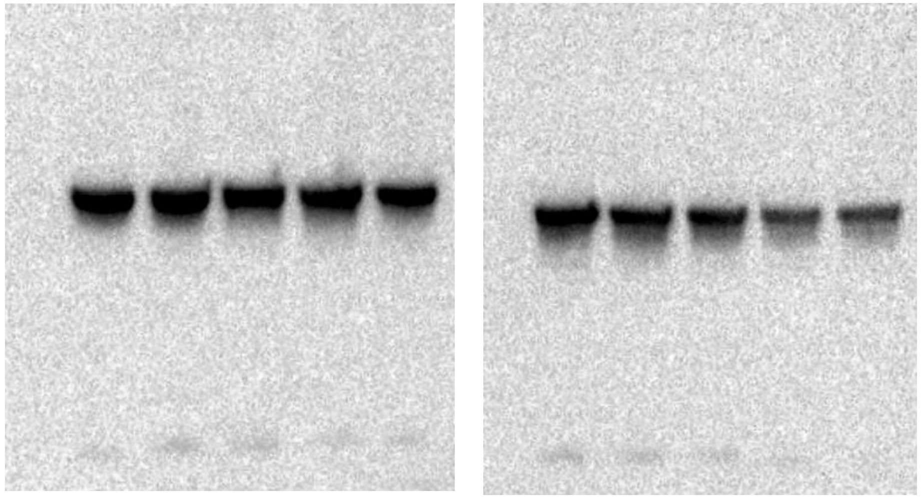

68kDA

Control FNDC5-KO

β-Actin

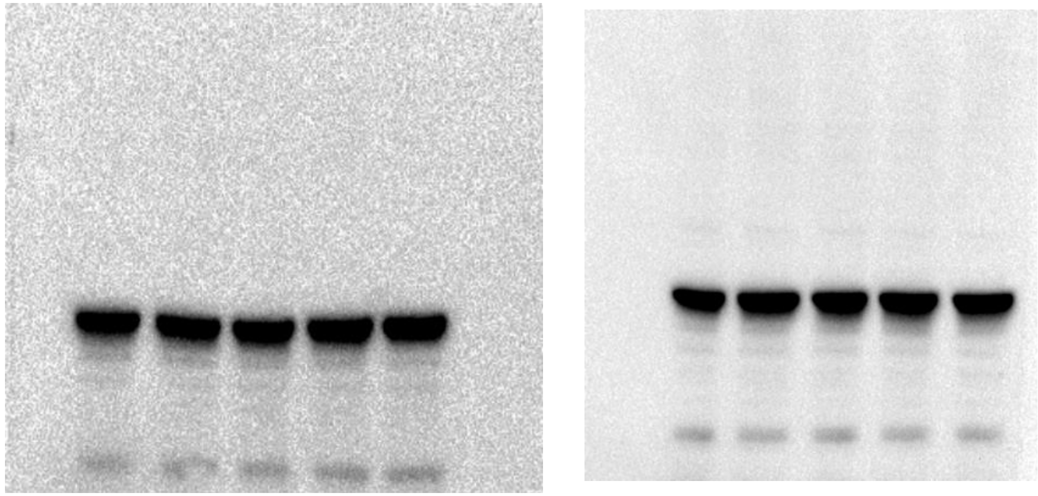

43kDA

Full Western Blots for Fig 7k

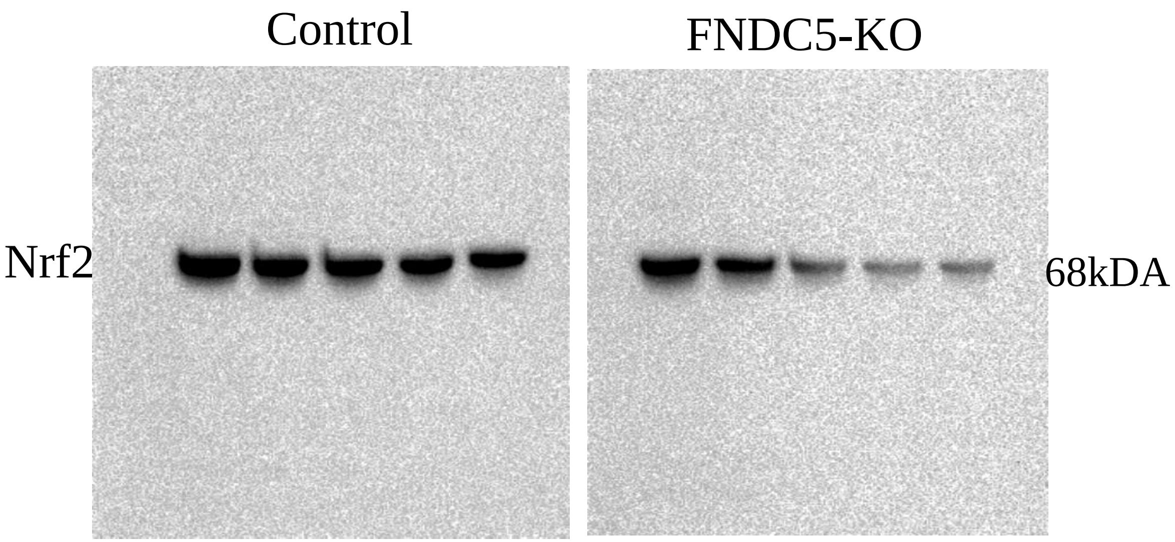

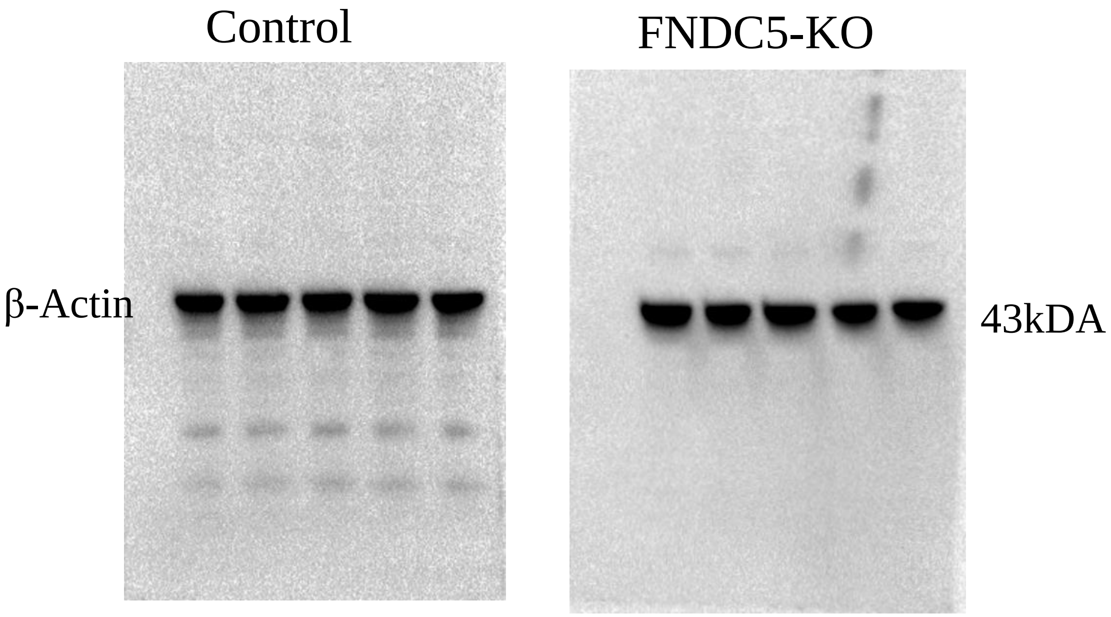

Full Western Blots for Fig 7m

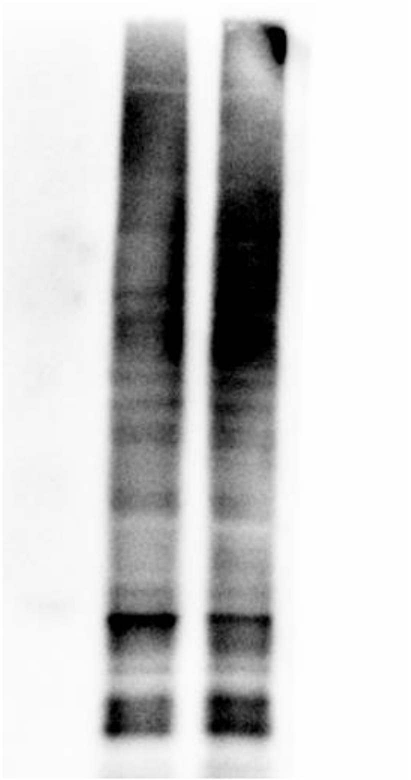

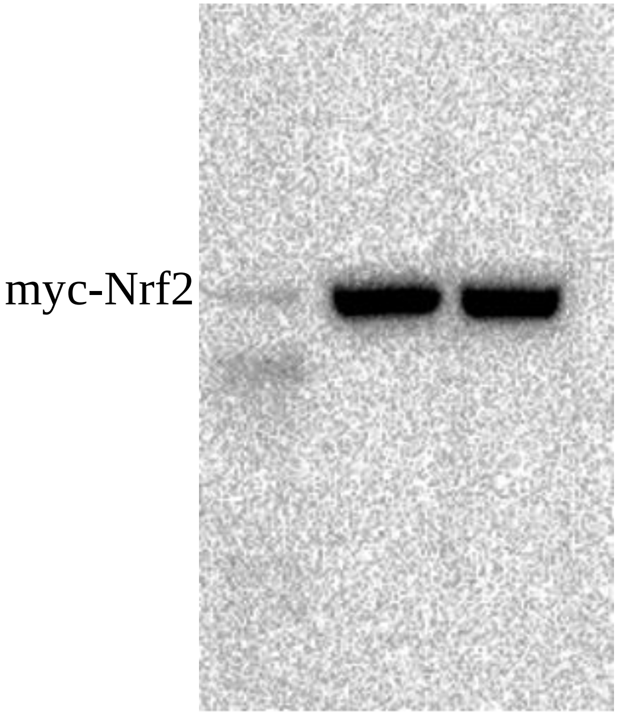

Full Western Blots for Fig S2 a

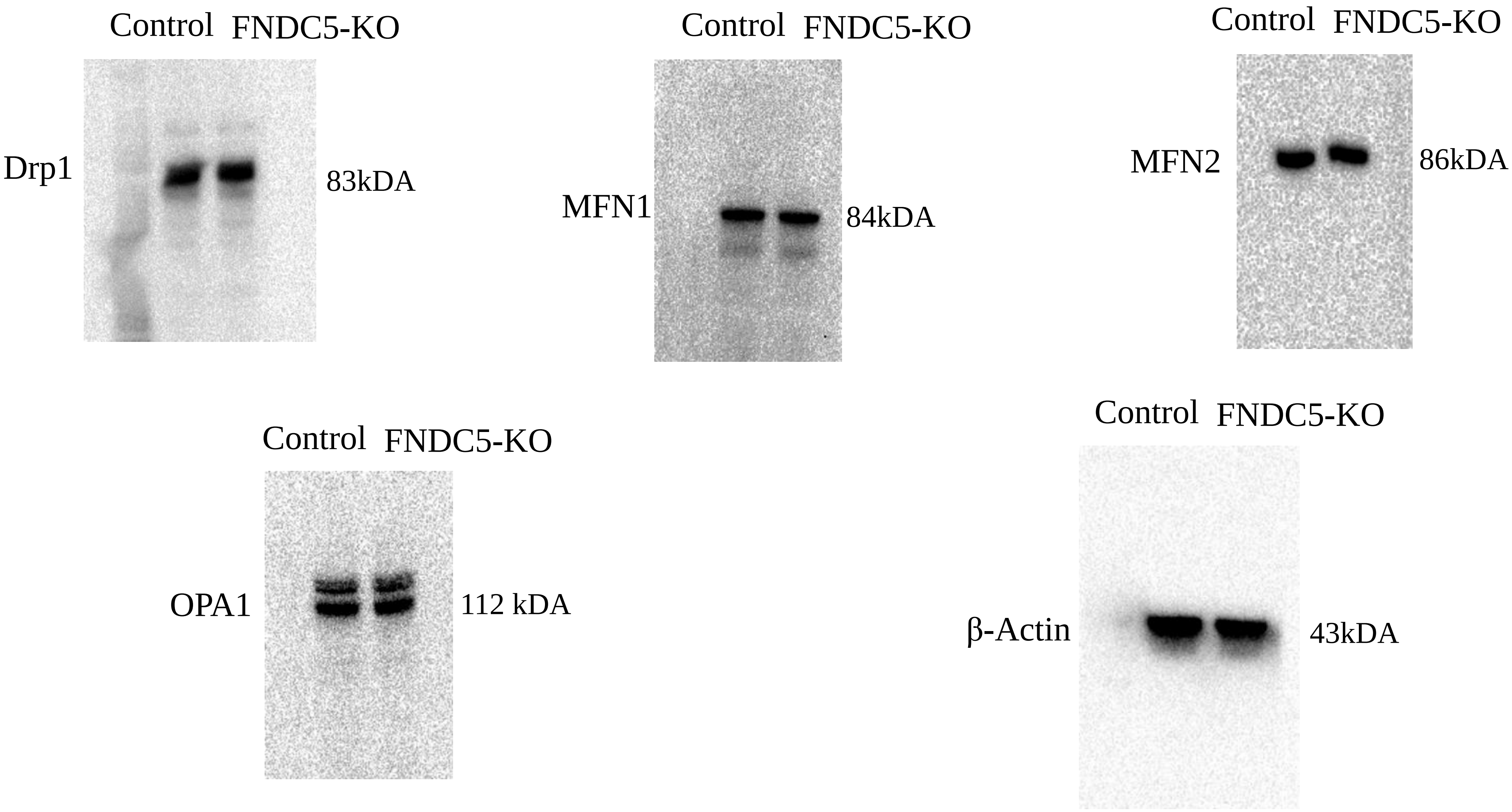

Full Western Blots for Fig S2 f

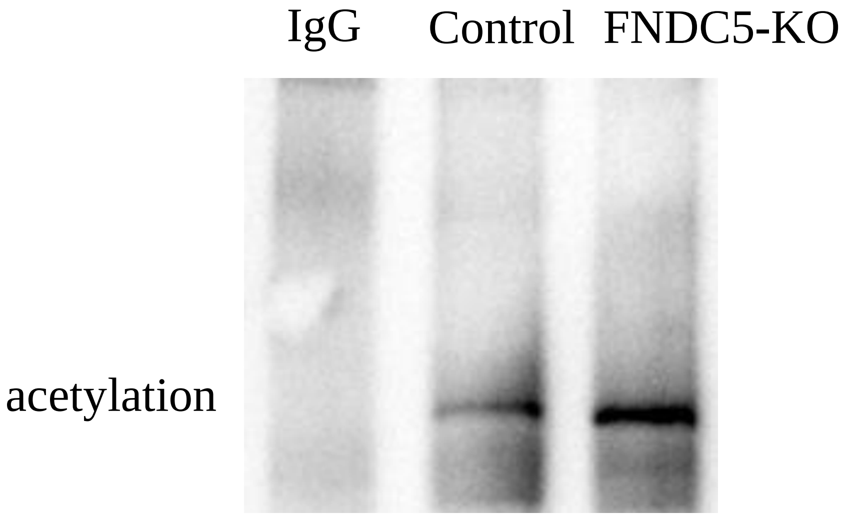

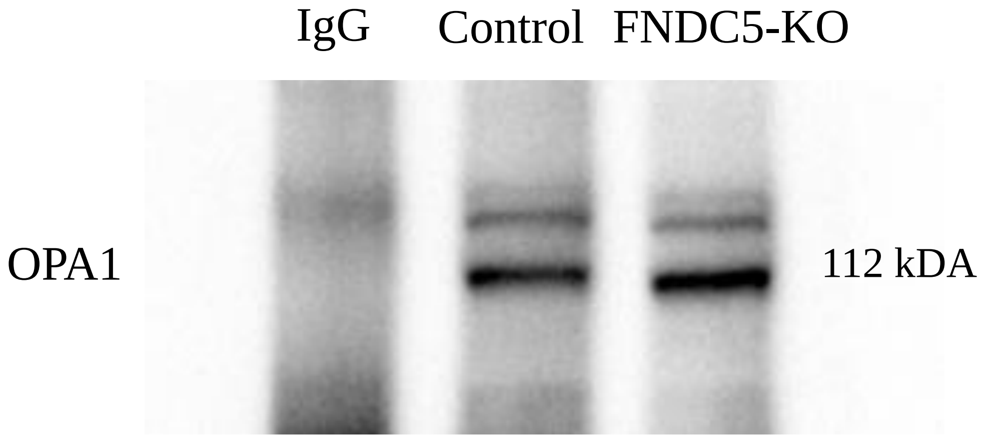

Full Western Blots for Fig S2 g

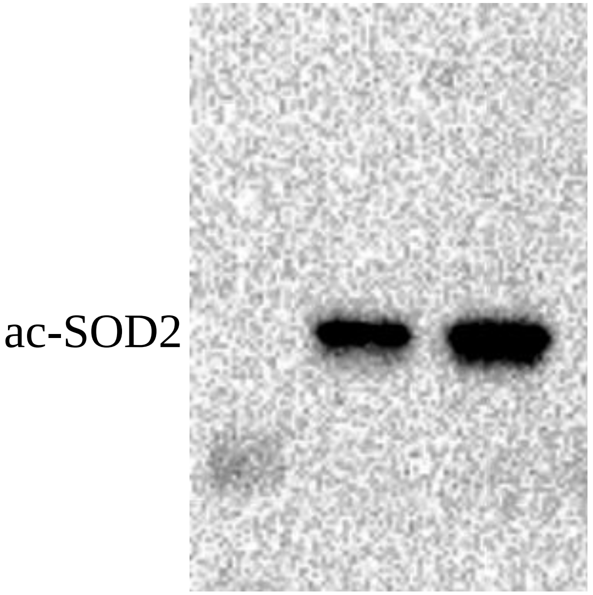

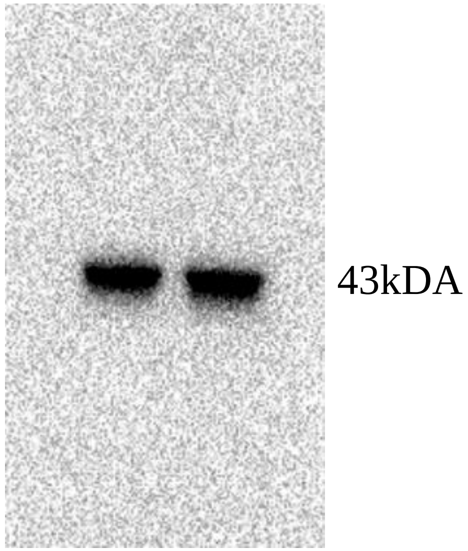

Full Western Blots for Fig S1 A

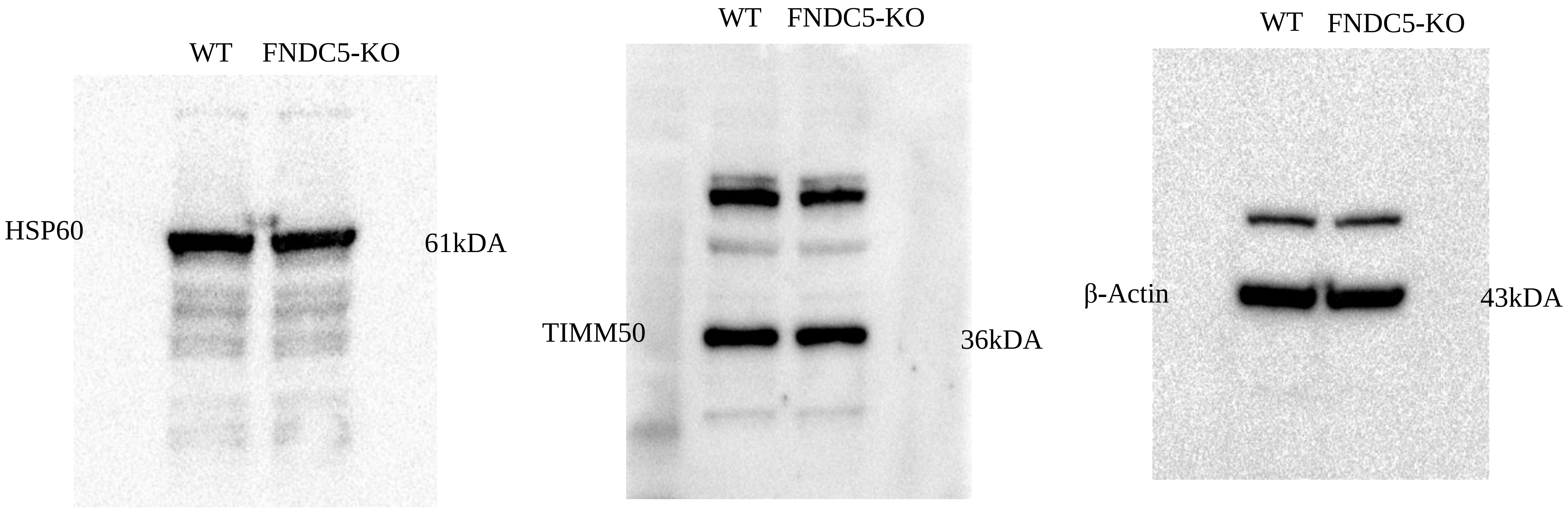

Supplement: Supplementary file 2 — Original Data File [file 41419_2024_6748_MOESM2_ESM.pdf]
